# Biomimetic ADME Profiling of Multifunctional Biphenylalkoxyamine-Related Histamine H_3_ Receptor Ligands with Anti-Alzheimer Potential

**DOI:** 10.3390/membranes16100315

**Published:** 2026-09-25

**Authors:** Stefan Simić, Dorota Łażewska, Predrag Kalaba, Judith Wackerlig-Damle, Thierry Langer

**Affiliations:** 1 Department of Pharmaceutical Sciences, University of Vienna, Josef-Holaubek-Platz 2, 1090 Vienna, Austria; 2 Vienna Doctoral School of Pharmaceutical, Nutritional and Sport Sciences, University of Vienna, Josef-Holaubek-Platz 2, 1090 Vienna, Austria; 3 Chair of Chemical Technology and Biotechnology of Drugs, Jagiellonian University Medical College, Medyczna Str. 9, 30-688 Kraków, Poland; 4 The Florey Institute of Neuroscience and Mental Health, 30 Royal Parade, Parkville, VIC 3052, Australia

**Keywords:** drug permeability, biomimetic membranes, immobilized artificial membrane (IAM) chromatography, drug–membrane interactions, blood–brain barrier permeability, intestinal absorption, phospholipid affinity, ADME profiling, CNS drug development, histamine H_3_ receptor ligands

## Abstract

Multifunctional histamine H_3_ receptor (H_3_R) ligands with cholinesterase inhibitory activity are being explored as potential Alzheimer’s disease therapeutics, but their interactions with biological membranes and associated ADME properties require parallel evaluation. Here, six structurally related alkoxyamine H_3_R ligands were characterized using an HPLC-based biomimetic workflow centered on immobilized artificial membrane (IAM) chromatography to assess phospholipid affinity, estimate passive blood–brain barrier permeability and human intestinal absorption. IAM measurements were complemented by HSA/AGP plasma protein binding, biomimetic distribution descriptors, plasma stability, chromatographic lipophilicity at pH 7.4 and solubility in JP1 (pH 1.2), JP2 (pH 6.8), and phosphate buffer (pH 7.4). The IAM models classified all six ligands as CNS-positive and predicted high intestinal absorption, while CHI_IAM_ values indicated strong membrane affinity. After 24 h, 77.5–89.5% and 62.2–80.5% parent compound remained in human and rat plasma, respectively. Solubility remained measurable across all three media. Integrated consideration of membrane-related ADME properties and previously reported pharmacology prioritized compounds (**6**), (**5**) and (**2**). Compound (**6**) showed the strongest overall potency–developability balance, although the biomimetic membrane predictions require confirmation in direct permeability, transporter, and in vivo pharmacokinetic studies.

## 1. Introduction

Alzheimer’s disease (AD) is a progressive neurodegenerative disorder characterized by gradual cognitive decline and memory impairment, including deficits in language, learning, visuospatial abilities, and executive function, and it remains the most common cause of dementia in the elderly population [1,2,3,4]. The multifactorial nature of AD, involving cholinergic dysfunction, amyloid-β and tau pathology, oxidative stress, mitochondrial impairment, and neuroinflammation, limits the efficacy of classical single-target therapeutic strategies and has stimulated interest in multitarget-directed ligands (MTDLs) [3,5,6,7].

Among the currently used symptomatic treatments, donepezil and rivastigmine enhance cholinergic neurotransmission by inhibiting acetylcholinesterase (AChE) or both AChE and butyrylcholinesterase (BuChE) respectively, but their clinical benefit remains modest and does not halt disease progression [8,9,10,11,12,13]. The continuing need for more effective anti-AD agents has therefore encouraged the development of multifunctional ligands capable of simultaneously modulating multiple disease-relevant targets [3,7,14,15,16].

Histamine H_3_ receptors (H_3_R) are presynaptic G-protein-coupled receptors predominantly expressed in brain regions associated primarily with neurotransmitter regulation, cognition and wakefulness [2,3,4,17]. Antagonists or inverse agonists of H_3_R can enhance the release of several neurotransmitters relevant to cognition, including acetylcholine, dopamine, noradrenaline, and histamine, making H_3_R a particularly attractive target in the search for novel anti-Alzheimer agents. Mechanistically, H_3_R antagonism or inverse agonism can facilitate presynaptic release of acetylcholine and other cognition-related neurotransmitters, whereas BuChE inhibition acts downstream by limiting acetylcholine (ACh) hydrolysis [14,18,19]. Combining these activities therefore addresses complementary steps in cholinergic signaling rather than two redundant targets. This provides a pharmacological rationale for the multitarget design, particularly as BuChE can assume a greater relative role in cholinergic hydrolysis during Alzheimer’s disease progression [1,4,18,19,20,21,22]. In this context, multitarget ligands that combine H_3_R modulation with cholinesterase inhibition or additional enzyme-targeting properties have emerged as particularly promising candidates for central nervous system (CNS) drug development aimed at mitigating the symptoms of Alzheimer’s disease [17,20,23,24].

The structurally related alkoxyamine H_3_ receptor ligand derivatives investigated in the present study originate from a previously reported series of multifunctional H_3_R ligands developed as potential anti-Alzheimer agents [17,20,23,24]. In the parent study, systematic structural variation of the cyclic amine moiety, alkyl linker length and aromatic substitution pattern yielded compounds with high affinity for H_3_R together with sub-micromolar inhibition of BuChE. Selected analogues also displayed activity toward monoamine oxidase B (MAO-B) as well as beneficial effects in an in vivo memory model [17,20,23,24].

However, for CNS-active compounds, favorable pharmacological activity alone is not sufficient, as successful development of novel drug candidates also depends on an appropriate absorption, distribution, metabolism, and excretion (ADME) profile [25,26,27,28,29]. Properties such as lipophilicity, blood-brain barrier permeability (BBB), human intestinal absorption (HIA), plasma protein binding (PPB), tissue distribution, plasma stability and aqueous solubility all influence whether a biologically active ligand can achieve sufficient exposure at its site of action [25,26,27,28,29,30,31].

HPLC-based biomimetic methods offer practical high-throughput tools for the early evaluation of these parameters, such as: immobilized artificial membrane (IAM) chromatography, which mimics phospholipid interactions and is therefore useful for estimating passive BBB permeability, intestinal absorption and phospholipid affinity, whereas stationary phases based on human serum albumin (HSA) and α1-acid glycoprotein (AGP) enable rapid estimation of plasma protein binding under physiologically relevant conditions [27,28,32,33,34]. When combined with HPLC-derived chromatographic lipophilicity at pH 7.4, plasma stability assessment, solubility measurements and established biomimetic distribution models for tissue distribution and brain tissue binding, these approaches provide a multidimensional view of compound behavior using relatively rapid and sample-sparing experimental workflows [25,31,35].

Therefore, the aim of the present study was to characterize the physicochemical and biomimetic ADME profile of six structurally related alkoxyamine H_3_R ligands previously reported as multifunctional candidates with potential relevance for CNS disorders and Alzheimer’s disease. The selected compounds include biphenyl, naphthalene, xanthone and cyanobiphenyl analogues that retain H_3_R affinity while displaying complementary biological activities, including cholinesterase inhibition, MAO-B inhibition, anticonvulsant effects, and beneficial outcomes in experimental memory-impairment models. Particular attention was devoted to evaluating their CNS developability in the context of H_3_R-targeted multitarget drug design through assessment of the ADME-related parameters described above, in order to determine whether these pharmacologically active scaffolds are also supported by a favorable biomimetic and physicochemical profile [17,20,25,31,32,36].

## 2. Materials and Methods

### 2.1. Test and Standards Compounds

The six investigated structurally related alkoxyamine H_3_ receptor ligands (Figure 1) share a common pharmacophoric arrangement consisting of a lipophilic aromatic domain connected through an alkyl ether linker to a protonatable cyclic amine. Structural variation within the series concerns the identity and topology of the aromatic domain, linker length, and cyclic amine. Compounds (**1**), (**3**), and (**4**) are biphenyloxyalkyl azepane derivatives with *ortho*-, *meta*-, and *para*-substitution patterns, respectively. (**2**) is a naphthyloxy piperidine derivative and therefore is not classified as a biphenyl analogue. (**5**) contains a xanthone scaffold, whereas compound (**6**) is a cyanobiphenyl derivative bearing a nitrile substituent.

These compounds were selected from the biphenylalkoxyamine derivatives and structurally related analogues series used in the study as multifunctional histamine H_3_R ligands with cholinesterase inhibitory activity. Their main advantage was not only their affinity for H_3_ receptors, but also their preferential inhibition of BuChE over AChE, which is relevant as BuChE becomes increasingly important in cholinergic neurotransmission during Alzheimer’s disease progression. Within this series, azepane derivatives were particularly advantageous, showing sub-micromolar BuChE IC_50_ values, potent MAO-B inhibition and in vivo activity in a scopolamine-induced memory impairment model [17,20,23,24].

The selected compounds (Table 1) represent non-imidazole histamine H_3_ receptor ligands built around a common pharmacophoric arrangement, in which a protonatable cyclic amine is connected through an alkyl ether linker to a lipophilic aromatic domain. Variation of this aromatic region produced distinct biological profiles across the series. Compounds (**1**), (**3**), and (**4**) are biphenyloxyalkyl azepane derivatives with *ortho*-, *meta*-, and *para*-substitution patterns, respectively. These compounds showed moderate-to-good *h*H_3_R affinity and a clear preference for BuChE inhibition over AChE, with *eq*BuChE IC_50_ values in the submicromolar range. Among them, (**3**) showed the most potent *eq*BuChE inhibition, whereas (**4**) displayed the weakest *h*H_3_R affinity in the selected set. (**2**), a naphthyloxy piperidine derivative, retained good *h*H_3_R affinity and displayed anticonvulsant activity in maximal electroshock and 6 Hz psychomotor-seizure models, indicating a pharmacological profile distinct from the cholinesterase-oriented biphenyl derivatives. (**5**), a xanthone-based azepane derivative, combined nanomolar *h*H_3_R affinity with potent AChE inhibition, moderate BuChE inhibition, *h*MAO-B inhibition, and in vivo memory-enhancing and analgesic effects. (**6**), the cyanobiphenyl derivative, exhibited the highest *h*H_3_R affinity among the compounds listed and showed low-micromolar inhibition of AChE, BuChE, and *h*MAO-B, together with beneficial activity in a scopolamine-induced memory-impairment model. Overall, these data indicate that the common H_3_R-directed pharmacophore tolerates substantial modification of the aromatic moiety, while the nature and substitution pattern of this region strongly influence cholinesterase inhibition, MAO-B activity and the resulting in vivo pharmacological effects.

The stock solutions of the investigated compounds were prepared in dimethyl sulfoxide (DMSO) (Merck, Darmstadt, Germany) at a concentration of 10 mM. Prior to analysis, these solutions were diluted with the mobile phase to 250 µM working solutions for the use in biomimetic chromatography assays and HPLC-derived chromatographic lipophilicity measurements at pH 7.4.

All six investigated compounds were evaluated in their oxalate salt forms throughout the experimental workflow. The aqueous pH was assay-specific and followed the corresponding experimental protocol: pH 7.0 for the BBB IAM assay, pH 5.5 for the HIA IAM assay, pH 7.4 for CHI_IAM_ and HSA/AGP plasma-protein-binding experiments, and pH 7.4 for the C_18_ chromatographic lipophilicity determination. Solubility was measured in JP1 at pH 1.2, JP2 at pH 6.8 and in phosphate buffer at pH 7.4. Thus, the ionization state during each measurement was governed by the assay medium.

Standard compounds used in biomimetic chromatography and chemicals for buffer preparation were purchased from Sigma Aldrich (Schnelldorf, Germany) in the highest available purity and from Gall Pharma (Judenburg, Austria) as pharmaceutical reference standards. Phosphoric acid was obtained from Carl Roth (Karlsruhe, Germany). Solvents, including acetonitrile, 2-propanol, water and methanol, were purchased from Merck (Darmstadt, Germany) in HPLC or LC-MS grade, depending on the analyses performed.

Samples for BBB penetration and intestinal absorption determination were prepared by mixing the working solutions with Dulbecco’s phosphate-buffered saline (Sigma-Aldrich, Schnelldorf, Germany) containing 20% acetonitrile (1:1, *v*/*v*) to obtain a final concentration of 250 µM.

For plasma protein binding experiments, samples were prepared by combining the working solutions with a mixture of 50 mM ammonium acetate (Sigma-Aldrich, Schnelldorf, Germany) buffer (pH 7.4) and 2-propanol (Merck, Darmstadt, Germany) (1:1, *v*/*v*), resulting in a final concentration of 250 µM.

The standard mixture for HPLC-derived chromatographic lipophilicity determination at pH 7.4 was prepared by dissolving 2 mg of triphenylene (Sigma-Aldrich, Schnelldorf, Germany) and 0.2 mL of toluene (Merck, Darmstadt, Germany) in methanol (Merck, Darmstadt, Germany) to a final volume of 20 mL. Samples (concentration of 250 µM) for HPLC analysis were obtained by mixing the working solutions with the standard mixture.

Samples in the experimental determination of chromatographic hydrophobicity index (CHI) were prepared by mixing the working solutions with a mixture of 50 mM ammonium acetate buffer (pH 7.4) and acetonitrile (Merck, Darmstadt, Germany), resulting in a final concentration of 250 µM.

For the solubility assay, three media were used: JP1, JP2 and a phosphate buffer (pH 7.4). JP1 (artificial gastric fluid, pH 1.2; 35 mM NaCl and 84 mM HCl) was prepared by dissolving 2.0 g of sodium chloride (Sigma-Aldrich, Schnelldorf, Germany) and adding 7.0 mL of HCl (Merck, Darmstadt, Germany), followed by dilution with water to a volume of 1000 mL. The JP2 (artificial intestinal fluid, pH 6.8; 50 mM phosphate buffer) was prepared by mixing 250 mL of 200 mM KH_2_PO_4_ (Sigma-Aldrich, Schnelldorf, Germany) with 118 mL of 200 mM NaOH (Merck, Darmstadt, Germany) and diluting to a final volume of 1000 mL with purified water. A phosphate buffer at pH 7.4 (200 mM) was prepared by mixing appropriate amounts of 200 mM K_2_HPO_4_ (Sigma-Aldrich, Schnelldorf, Germany) and 200 mM KH_2_PO_4_ and adjusting the pH to 7.4.

### 2.2. Blood–Brain Barrier (BBB) Permeability and Human Intestinal Absorption (HIA) Assessment by IAM Chromatography 

Chromatographic experiments were performed using a Nexera XR UHPLC system (Shimadzu Corporation, Tokyo, Japan) equipped with an IAM.PC.DD.2 column (100 × 4.6 mm, 10 μm; Regis Technologies, Morton Grove, IL, USA). The mobile phase consisted of HPLC-grade acetonitrile and Dulbecco’s phosphate-buffered saline (DPBS). For blood–brain barrier permeability assessment, the mobile-phase composition was 20% acetonitrile and 80% DPBS adjusted to pH 7.0 ± 0.05 using sodium hydroxide. For the intestinal absorption studies, the pH of the DPBS was adjusted to 5.5 ± 0.05 using 2N hydrochloric acid. The flow rate of 1.0 mL/min and column temperature of 37 °C were maintained. The injection volume was set at 5 µL. Detection was performed using a diode array detector over a wavelength range of 190 to 400 nm.

The resulting IAM retention (capacity) factor (*k_IAM_*) was determined by Equation (1), where t_R_ represents the retention time of the analyzed compound and t_0_ representing the void volume, which was determined by injecting 5 µL of uracil (100 µM).

(1) $$k_{IAM}=\frac{t_{R}-t_{0}}{t_{0}}$$

To account for the influence of molecular size on diffusion through the BBB, permeability calculations incorporated molecular weight-dependent corrections, as previously described in IAM-based permeability models [28,29,38]. The permeability descriptor (P_m_) was calculated using a power function relationship between *k_IAM_* and the molecular weight, as previously reported and expressed with the following equation [39]:

(2) $$\mathrm{Pm}=\frac{k_{IAM}}{{MW}^{4}}\times 10^{10}$$

A quantitative structure–activity relationship (QSAR) model (Equation (3)) for the prediction of the percentage of human intestinal absorption (%HIA) was applied based on a previously reported multiple linear regression model [39]. The model incorporates physicochemical descriptors, including polar surface area (PSA) and logarithm of the partition coefficient, together with interaction terms involving experimentally determined *k*_IAM_ values at the pH of 5.5 [39]. The relevance of these physicochemical descriptors for intestinal drug absorption has been widely reported in the literature [27,40]. The physicochemical descriptors used in determining of the human intestinal absorption were calculated with the use of BIOVIA Dassault Systèmes Draw 2022 (Dassault Systèmes, San Diego, CA, USA).

(3) %HIA = 122.366 (±4.527) − 0.417 (±0.035) × PSA − 5.269 (±1.584) × logP + 0.092 (±0.019) × (PSA × logP) − 9.882 (±0.641) × (1/*k_IAM_* · logP) *n* = 20, R = 0.988, R^2^ = 0.975, R^2^_adj_ = 0.969, s = 4.038, F = 147.221

The %HIA values generated by this published QSAR are model-based estimates rather than direct measurements of intestinal permeability. The logP term is the computational descriptor required by the original model, whereas *k_IAM_* was measured experimentally at pH 5.5. For these ionizable tertiary amines, the model does not constitute a direct determination of pH-dependent logD and does not explicitly resolve transporter effects.

### 2.3. HPLC-Derived Chromatographic Lipophilicity Estimation at pH 7.4

Samples for HPLC-derived chromatographic lipophilicity estimation at pH 7.4 were analyzed using a Shimadzu Prominence LC-20AD XR liquid chromatography system (Shimadzu Corporation, Tokyo, Japan) equipped with a Shim-pack GIST C_18_ column (3 µm, 4.6 × 50 mm, Shimadzu Corporation, Tokyo, Japan). Chromatographic separation was carried out using a gradient elution consisting of mixture of 0.01 M phosphate buffer (pH 7.4) and methanol (1:1, *v*/*v*) (solvent A) and HPLC-grade methanol (solvent B) (Merck KGaA, Darmstadt, Germany). The gradient increased from 0% to 80% solvent B over 8 min, followed by an isocratic hold at 80% B for 4 min. The flow rate was set to 1.2 mL/min, and the column oven temperature was maintained at 40 °C. The injection volume of the samples (250 µM) was 5 µL. Retention times of reference compounds and analytes were determined by peak integration at a wavelength of 254 nm. The chromatographic lipophilicity estimates, reported as experimental logP values for continuity with the published calibration method, were calculated using toluene and triphenylene as reference standards according to the method (Equation (4)), as previously described [36]:

Because the investigated compounds contain ionizable tertiary amines and were introduced as oxalate salts into a mobile phase buffered at pH 7.4, these HPLC-derived values should be interpreted as method-specific apparent chromatographic lipophilicity estimates under pH 7.4 conditions.

(4) $$logP=\frac{\left({logP}_{Toluene}-{logP}_{Triphenylene}\right){\times Rt}_{Compound}+{Rt}_{Toluene}\times {logP}_{Triphenylene}-{Rt}_{Triphenylene}\times {logP}_{Toluene}}{{Rt}_{Toluene}-{Rt}_{Triphenylene}}$$

The in silico logP values were determined with the use of ACD/ChemSketch 2021.2.1 (ACD/Labs, Toronto, ON, Canada), BIOVIA Dassault Systèmes Draw 2022 (Dassault Systèmes, San Diego, CA, USA), ChemDraw 23 (Revvity Signals Software, Waltham, MA, USA) for Windows 11 and SwissADME (Swiss Institute of Bioinformatics, Ecublens, Switzerland) [41].

### 2.4. Chromatographic Hydrophobicity Index on Immobilized Artificial Membrane

The samples for the determination of the chromatographic hydrophobicity index on immobilized artificial membrane (CHI_IAM_) were analyzed using a Shimadzu Prominence LC-20AD XR liquid chromatography system (Shimadzu Corporation, Tokyo, Japan) equipped with an IAM.PC.DD.2 column (3 × 4.6 mm, 12 μm; Regis Technologies, Morton Grove, IL, USA). The experiment was performed with the use of a gradient elution with 50 mM ammonium acetate buffer (pH 7.40 ± 0.05) (solvent A) and HPLC-grade acetonitrile (solvent B) (Merck KGaA, Darmstadt, Germany). The gradient method was applied as follows: 0% solvent B at 0–3 min, 0–70% solvent B, 3–5 min 70–80% solvent B, 5–7 min 80–100% solvent B, 7–8 min 100% solvent B.

The flow rate was set to 0.5 mL/min, while the column oven was maintained at a temperature of 25 °C. The injection volume was 5 µL (250 µM). For the retention time determination, detection wavelengths were set between 190 and 400 nm, depending on the maximum absorbance of each compound. The dead time was once again determined by injecting 5 µL uracil (100 µM) along with the sample.

Five acetonitrile (B) levels were chosen for each reference compound based on its retention characteristics. Chlorothiazide was measured at 5, 10, 15, 20, and 25% B; paracetamol, propyphenazone, propyl p-hydroxybenzoate, valerophenone, and 2,3-dichlorophenol at 15, 20, 25, 30, and 35% B; octanophenone at 30, 35, 40, 45, and 50% B; and tamoxifen at 50, 55, 60, 65, and 70% B. Each isocratic mobile-phase composition was analyzed in triplicate. The logk_IAM_ value was calculated using Equation (5):

(5) $$logk_{IAM}=log\frac{t_{R}-t_{0}}{t_{0}}$$

where t_0_ is the dead time and tᵣ is the retention time of the standard. The log*k_IAM_* values were plotted against the five different applied acetonitrile concentrations. The intercept (log k_xx_) and slope (S) were used to calculate the isocratic hydrophobicity index (φ_0_) using Equation (6).

(6) $$\varphi_{0}=\frac{{logk}_{w}}{S}$$

The CHI_IAM_ value was calculated with slope A and intercept B, according to the equation:

(7) CHI_IAM_ = At_R_ + B

The CHI_IAM_ values obtained from gradient measurements were converted to logK_IAM_ and logk_IAM_ values using the following equations [34]:

(8) log*k_IAM_* = 0.046 ×CHI_IAM_ + 0.42

(9) logK_IAM_ = 0.29 × e^logk(IAM)^ + 0.70

### 2.5. Plasma Protein Binding (PPB) Analysis Using HSA and AGP Biomimetic HPLC Columns

All measurements were performed using a Shimadzu Prominence LC-20AD XR liquid chromatograph (Shimadzu Corporation, Tokyo, Japan) equipped with a photodiode-array detector. The plasma protein binding assay was carried out on two biomimetic chromatography columns: CHIRALPAK-HSA (5 µm, 3 × 50 mm, Daicel Inc., Tokyo, Japan), representing the binding to the human serum albumin and CHIRALPAK-AGP (5 µm, 3 × 50 mm, Daicel Inc., Tokyo, Japan), which mimics the binding to the alpha-1-acid glycoprotein. The optimized gradient was applied: 0–3 min: 0% B; 3–10 min: 30% B [8]. Solvent A was a 50 mM ammonium acetate buffer adjusted to a physiological pH of 7.4 ± 0.05., while the solvent B consisted of 2-propanol. The flow rate was maintained at 1.0 mL/min, and the column temperature was set to 30 °C. A reduced flow rate, compared to the original method described by Valko et al., was employed to prolong column lifetime, as higher flow rates would approach the maximum allowable operating pressure of the column [32]. The injection volume of the samples (250 µL) was 5 µL.

For column calibration, literature plasma protein binding (%PPB) values were converted into linear free energy–related logk values (logarithm of the apparent affinity constant) using following equation [32]:

(10) $$logk=log\frac{\%PPB}{101-\%PPB}$$

The logarithm of the retention times (log t_R_), obtained from triplicate injections of each compound, was plotted against the corresponding log*k* values. The slope and intercept of the resulting linear relationship were then used to convert gradient retention times into logk values for the analyzed compounds. Based on these calculated log*k* values, the percentage of protein binding was subsequently determined using Equation (11) [32].

(11) $$\%Binding=\frac{101\times 10^{logk}}{1+10^{logk}}$$

For calibration of the HSA column, thirteen reference compounds with well-characterized plasma protein binding properties were selected, covering a wide PPB range from 19.0% (cimetidine) to 99.8% (diclofenac). For the AGP column, seven standard compounds were used, spanning a wide PPB range from 3.2% (paracetamol) to 87.0% (nicardipine).

For biomimetic distribution model estimation, the logk values obtained from the HSA column were further transformed into logK_HSA_ values to establish a linear correlation with the linear free energy–related partition coefficient, according to the following equation [34]:

(12) logK_HSA_ = e^log^*^k^*^(HSA)^

### 2.6. Biomimetic Distribution Models

The combination of HSA and IAM retention data was utilized to estimate pharmacokinetic parameters including volume of distribution (V_d_), unbound volume of distribution (V_du_), and brain tissue binding (BTB), using established biomimetic distribution models. The corresponding models are described in Equations (13)–(16) [26,34].

(13) logV_d_ = 0.44 × logK_IAM_ − 0.22 × logK_HSA_ − 0.62

(14) logV_du_ = 0.23 × logK_HSA_ + 0.43 × logK_IAM_ − 0.72

(15) log*k*_BTB_ = 1.29 × log*k*_IAM_ + 1.03 × log*k*_HSA_ − 2.37

(16) $$\%BTB=\frac{100\times 10{logk}_{BTB}}{1+10{logk}_{BTB}}$$

After inverse transformation, model-derived V_d_ and V_du_ values are expressed on a body-mass-normalized basis (L/kg). These empirical HSA/IAM relationships are intended for comparative estimation and can become highly extrapolative when compounds fall outside the central property range represented by the calibration data.

### 2.7. Plasma Stability Assay

Ultra-high-performance liquid chromatography–mass spectrometry analyses were performed using an UltiMate 3000 RSLC system (Thermo Fisher Scientific, Germering, Germany) coupled to a maXis HD ESI-Qq-TOF mass spectrometer (Bruker Corporation, Bremen, Germany). Separation was conducted on a Kinetex Phenyl-Hexyl column (2.6 µm, 2.1 × 50 mm, Phenomenex, Torrance, CA, USA) equipped with an appropriate guard column. The electrospray ionization (ESI) source was operated under the following conditions: capillary voltage of 3.5 kV, nebulizer pressure at 0.8 bar (N_2_), dry gas flow rate of 7.0 L/min (N_2_), and a drying temperature of 200 °C. Mass spectra were acquired in positive ion full-scan mode over an m/z range of 50–2500. The chromatographic separation was performed using gradient elution with methanol (MeOH) (Merck KGaA, Darmstadt, Germany) as solvent B and water containing 0.1% formic acid (FA) (98%, Carl Roth GmbH + Co. KG, Karlsruhe, Germany) as solvent A. All solvents used were LC-MS grade. The gradient program was as follows: 20% solvent B from 0 to 1 min, increased to 70% solvent B at 3 min and to 95% solvent B at 6 min, followed by a washing phase at 95% solvent B for 3 min and subsequent column re-equilibration at 20% solvent B for 3.5 min. The LC-MS measurements were performed at a flow rate of 500 µL/min with the oven temperature set at 37 °C. Injection volume for the samples was 2 µL.

Plasma stability was evaluated by preparing each compound in triplicate. Pooled human plasma was thawed at room temperature, vortexed, and pre-equilibrated at 37 °C for 3 min using an Eppendorf MixMate™ (Eppendorf, Hamburg, Germany). Subsequently, 10 µL of the stock solution was added to 490 µL of blank plasma to obtain a final concentration of 200 µM. Samples were vortexed for 20 s and incubated at 37 °C with gentle agitation (500 rpm) for predefined time intervals (0, 0.5, 1, 1.5, 2, 4, and 24 h). At each time point, 40 µL aliquots were withdrawn and immediately mixed with 100 µL of ice-cold methanol containing the internal standard (IS; losartan) at a ratio of 1:2.5 (*v*/*v*) to precipitate plasma proteins. Following thorough vortexing, samples were centrifuged at 14,000 × g at 4 °C for 15 min. An aliquot of 2 µL of the supernatant was subsequently injected into the LC–MS system for analysis. The same procedure was applied to both matrices. Blank pooled human plasma containing citrate was obtained from Biowest (Nuaillé, France), while blank pooled Sprague–Dawley rat plasma containing K_2_-EDTA was obtained from NeoBiotech (Nanterre, France).

At each incubation time, the triplicate analyte-to-internal-standard peak-area ratios were averaged. The mean response at each time point was then normalized to the corresponding mean response at 0 h, such that the normalized response represented the fraction of analyte remaining:

(17) $$R_{t}=\frac{C_{t}}{C_{0}}$$

where C_0_ is the mean analyte-to-internal-standard response at 0 h and C_t_ is the corresponding mean response at incubation time *t*. Apparent plasma degradation was evaluated by ordinary least-squares regression of the natural logarithm of the normalized mean response against time, assuming first-order kinetics [42,43].

(18) $$ln\left(\frac{C_{t}}{C_{0}}\right)=a-k_{obs}t$$

In this model, a is the fitted intercept and *k*_obs_ is the apparent first-order degradation rate constant. The *k*_obs_ value corresponds to the negative slope of the fitted regression line:

(19) $$k_{obs}=-\mathrm{Slope}$$

The apparent plasma half-life was calculated as:

(20) $$t_{1/2}=\frac{ln\left(2\right)}{k_{obs}}$$

Linear regression was performed using the seven mean time-point responses obtained at 0, 0.5, 1, 1.5, 2, 4 and 24 h. Goodness of fit was assessed using the coefficient of determination (R^2^), and the two-sided *p* value for the fitted slope was reported. Two-sided 95% confidence intervals for *k*_obs_ were calculated from the standard error of the fitted slope using the Student *t* distribution with 5 degrees of freedom:

(21) $${CI}_{95\%}\left(k_{obs}\right)=k_{obs}\pm t_{0.975,5}SE\left(k_{obs}\right)$$

When the lower confidence limit of *k*_obs_ was greater than zero, the corresponding 95% confidence interval for the apparent half-life was obtained by inverse transformation:

(22) $${CI}_{95\%}\left(t_{1/2}\right)=\left[\frac{ln\left(2\right)}{k_{obs,upper}},\frac{ln\left(2\right)}{k_{obs,lower}}\right]$$

When the confidence interval for *k*_obs_ included zero, the upper confidence limit of t_1/2_ was considered unbounded. In such cases, only the lower 95% confidence bound is reported (≥value). Because all apparent half-lives extended beyond the 24 h observation period, these estimates should be interpreted as comparative in vitro stability parameters rather than direct predictions of systemic exposure, dosing interval, or clinical elimination half-life.

### 2.8. Solubility Assay

The samples for solubility determination were analyzed using a Shimadzu Prominence LC-20AD XR chromatography system (Shimadzu Corporation, Tokyo, Japan) equipped with a photodiode-array detector. The HPLC system was fitted with a Shim-pack GIST C_18_ column (3 µm, 4.6 × 50 mm, Shimadzu Corporation, Tokyo, Japan) coupled with a suitable precolumn.

Analyses were performed using gradient elution with HPLC-grade water containing 0.1% phosphoric acid (solvent A) and HPLC-grade acetonitrile (solvent B) (Merck KGaA, Darmstadt, Germany). The gradient started at 5% solvent B, increased linearly to 90% B over 3 min, and was maintained at 90% B for 2.5 min. The column oven temperature was set to 40 °C, the flow rate was 1.0 mL/min, and the injection volume was 100 µL. Detection was carried out using a diode array detector over a wavelength range of 210–400 nm.

For sample preparation, 50 µL of 10 mM DMSO stock solutions were transferred into 2 mL Eppendorf tubes, followed by the addition of 450 µL of the respective medium, resulting in a total volume of 500 µL. The samples were vigorously shaken for 1 h using an Eppendorf MixMate™ (Eppendorf, Hamburg, Germany) at 3000 rpm. Subsequently, the samples were centrifuged at 14,000 rpm for 10 min. An aliquot of 50 µL of the supernatant was diluted with 100 µL of 50% acetonitrile and injected (100 µL) into the HPLC system.

For calibration, 10 µL of the 10 mM stock solution was diluted with 490 µL of 50% acetonitrile to obtain a 200 µM solution. Serial dilutions were then prepared to yield concentrations of 20, 2, and 0.2 µM. These four standards (200, 20, 2, and 0.2 µM) were analyzed to generate a four-point calibration curve.

Solubility was calculated based on the integrated chromatographic peak area using the following equation [35]:

(23) $$Solubility\left(µM\right)=Concentrationoftwo-folddilutedfiltrate\times 2$$

The capacity factor (*k*′) was calculated with the use of Equation (24), where t_0_ was once again determined by injecting 5 µL uracil (100 µM).

(24) $$k^{\prime }=\frac{t_{R}-t_{0}}{t_{0}}$$

## 3. Results and Discussion

### 3.1. Blood–Brain Barrier (BBB) Permeability and Human Intestinal Absorption (HIA) Based on IAM Chromatography

The IAM-based BBB permeability (Table 2) assessment indicated that all tested biphenylalkoxyamine and structurally related analogues were classified as CNS-positive (CNS+) within the IAM model, with P_m_ values ranging from 14.9 to 42.4, thus lying well above the previously established CNS+ threshold of 1.51 [39]. In comparison with the reference drugs, all compounds (**1**)–(**6**) showed significantly higher P_m_ values than both donepezil (P_m_ = 2.20) and rivastigmine (P_m_ = 4.46), which corresponds to higher IAM-model passive-permeability estimates [28]. As P_m_ used here within a classification model, these values were interpreted for CNS+/CNS- classification rather than for quantitative comparison of brain exposure with the reference drugs [39]. The determined k_IAM_ values were also consistently high, ranging from 19.37 to 45.32, with low CV values (0.19–3.62%), confirming good reproducibility and strong affinity of the investigated compounds for the phospholipid-mimetic IAM stationary phase [38,44,45].

Within the series, compound (**2**) showed the highest P_m_ value (42.4), whereas compound (**3**) exhibited the lowest value (14.9). Despite these differences, all derivatives were assigned to the CNS+ class by the IAM model. Since the P_m_ descriptor incorporates both membrane affinity and molecular weight, the particularly high value observed for (**2**) reflects a favorable balance between strong IAM interaction and relatively lower molecular weight within the series [28,46]. In comparison to (**2**), compound (**5**) showed the highest k_IAM_ value (45.32) within the set but a lower P_m_ value, showing that stronger IAM retention did not correspond to the highest P_m_ value. This consistent with the molecular-weight correction included in the P_m_ descriptor [47,48].

From a structural perspective, the CNS+ classification of compound (**1**)–(**6**) is consistent with their predominantly aromatic and lipophilic character combined with the presence of a basic amino moiety. Such a combination is compatible with interaction with the phospholipid-mimetic IAM phase [49,50]. At the same time, the lower P_m_ of (**3**) compared with the compounds (**1**), (**2**), and (**4**) suggests that subtle differences in aromatic substitution pattern and electron distribution may influence interaction with the IAM stationary phase. Compound (**5**), bearing a tricyclic aromatic moiety, displayed the highest *k*_IAM_ value, which is in good agreement with its extended hydrophobic surface and as such the strong membrane affinity. Compound (**6**), containing a -CN substituent, still retained a high P_m_ value, which shows that although the nitrile group increases polarity, this additional polarity did not prevent classification within the CNS+ range [50,51,52].

Donepezil and rivastigmine were retained in Table 2 as reference compounds for the IAM assay. However, because the BBB prediction is based on a classification model, differences in P_m_ between the investigated compounds and the reference drugs were not used to infer relative brain exposure.

These results should be interpreted within the framework of a passive diffusion model. IAM chromatography reflects the interaction of compounds with phospholipid-like membranes and is therefore highly useful for estimating the passive BBB permeability potential of a screened compound, but the classification model does not account for active uptake or efflux transporters; therefore, CNS+ classification does not exclude potential transporter-mediated efflux in vivo [27,28,49,53,54]. Accordingly, the CNS+ classifications indicate compatibility with the passive membrane-permeation characteristics represented by the IAM model, but they do not establish net brain exposure. The reported in vivo CNS activity of selected analogues provides complementary pharmacological context: (**2**) showed anticonvulsant activity in MES and 6 Hz seizure models, (**5**) improved memory performance in dizocilpine-induced amnesia and showed analgesic effects in neuropathic-pain models, and (**6**) produced beneficial effects in scopolamine-induced memory impairment [17,23,24].

Therefore, the CNS+ classification reported here should be interpreted as an IAM-based indication of passive BBB permeability potential only and not as direct evidence that the compounds achieve brain exposure in vivo.

Regarding the intestinal absorption screening with the use of IAM chromatography (Table 3) all investigated biphenylalkoxyamine and structurally related analogues were predicted to exhibit high intestinal absorption, with %HIA values ranging between 80.9 and 85.0%. These results are close to the percentage of intestinal absorption of donepezil (85.8%) and somewhat higher than of rivastigmine (68.7%). This indicates a generally favorable HIA-model profile across the compound set but does not constitute a direct measurement of oral bioavailability. The relatively narrow range of %HIA values suggests that all compounds share physicochemical properties compatible with efficient intestinal permeation, despite some structural differences within the investigated set [27,55]. In particular, (**1**)–(**4**) showed absorption values above 82%, whilst (**1**) expressed the highest predicted %HIA at 85.0%. This overall trend is consistent with the used QSAR model, in which intestinal absorption is mainly governed by the interplay of polarity, lipophilicity, and IAM-derived membrane affinity [39,56,57].

From a structural point of view, compounds (**1**)–(**4**) share a closely related structure consisting of a biphenyl-type aromatic system, a nitrogen-containing cyclic ring, and an ether linker between these two parts and all showed very similar and high predicted %HIA values [57,58,59]. This suggests that positional modifications within the aromatic system did not substantially reduce the intestinal absorption potential of these derivatives. Their very low PSA value (12.47 Å^2^) is particularly favorable for passive membrane permeation, while the relatively high logP coefficient supports partitioning into the lipid membrane. Compound (**6**) exhibited the lowest %HIA value (80.9%) within the set, which is consistent with its somewhat higher polarity and lower membrane affinity compared with the other derivatives [52,56,60]. Overall, the dataset indicates that the predominantly lipophilic aromatic scaffold combined with limited polarity is rather highly favorable for intestinal absorption in this compound class [33,57,61,62].

In comparison with the reference drugs, the predicted absorption of compounds (**1**)–(**6**) yielded HIA-model values close to donepezil and above rivastigmine. These comparisons indicate similar model-predicted absorption within the published QSAR, but they do not demonstrate oral bioavailability. Because the model uses a calculated logP descriptor together with IAM retention measured at pH 5.5, direct intestinal-permeability and pH-dependent distribution studies are required before concluding that the compounds are orally absorbed [48,54,63].

### 3.2. HPLC-Derived Chromatographic Lipophilicity at pH 7.4 and In Silico logP Evaluation

Lipophilicity is a major determinant of ADME behavior due to its effect on membrane permeation, plasma protein binding, tissue distribution, metabolism, and clearance [64]. For CNS-active compounds, lipophilicity is highly important, since it strongly influences partitioning into biological membranes and therefore contributes to passive BBB penetration, as witnessed in the BBB IAM assay [48,51,65]. At the same time, the relationship is not simply linear: moderate lipophilicity is often favorable, since the disadvantage of the excessive lipophilicity is that it can increase nonspecific binding, decrease solubility and thus have an effect on bioassays, etc [53,63,66,67].

The in silico determined and experimentally obtained lipophilicity (Table 4) data show that compounds (**1**)–(**6**) are more lipophilic than the reference drugs donepezil and rivastigmine. The HPLC-derived chromatographic estimates obtained at pH 7.4 range from 4.81 to 6.55, whereas donepezil and rivastigmine gave values of 3.93 and 2.14, respectively. Within the investigated series, (**1**) was the most lipophilic, while (**6**) the least, but even the lowest HPLC-derived value remained above donepezil. These results indicate a pronounced hydrophobic character across the investigated compound set, consistent with their aromatic scaffolds, ether linkers, and cyclic tertiary amine-containing structures.

The high pH 7.4 chromatographic lipophilicity estimates are consistent with the strong IAM retention observed in the previous section, since compounds with greater hydrophobic surface area generally display stronger partitioning into lipid-like phases and biomimetic stationary phases [26,68]. At the same time, the comparatively high chromatographic lipophilicity of all investigated derivatives relative to donepezil and rivastigmine suggests that subsequent ADME interpretation should not focus only on permeability, but also on solubility, plasma protein binding, tissue distribution, metabolic stability, and the unbound fraction, because these parameters may become limiting for highly lipophilic CNS candidates [48,54].

From a structure-property perspective, the highest experimental lipophilicity was observed for (**1**), followed by (**4**) and (**3**), which is consistent with the presence of extended hydrophobic aromatic systems and a flexible alkoxyamine chain. (**2**) also remained strongly lipophilic, while (**5**), despite containing a more polar xanthone carbonyl group, retained a high logP value due to its rigid tricyclic aromatic surface. (**6**) showed the lowest logP within the series, in agreement with the presence of the -CN substituent, which increases polarity and partially counterbalances the hydrophobic contribution of the cyanobiphenyl scaffold.

The high lipophilicity of (**2**), (**5**), and (**6**) is compatible with their reported in vivo CNS-related activity, including anticonvulsant activity for the naphthyloxy analogue, pro-cognitive and analgesic effects for the xanthone derivative, and improvement of scopolamine-induced memory impairment for the cyanobiphenyl derivative. Nevertheless, in vivo efficacy should not be attributed to logP alone, because CNS activity also depends on receptor/enzyme potency, systemic exposure, brain penetration, efflux transport, metabolic stability and the unbound concentration at the site of action [17,24].

A difference was observed between the pH 7.4 HPLC-derived chromatographic and in silico lipophilicity estimates. For most of the synthesized derivatives, the calculated values from SwissADME, BIOVIA Draw 2022, ChemDraw 23, and ACD/ChemSketch 2021.2.1 generally followed the same ranking trend, but several methods tended to underestimate the HPLC-derived chromatographic values, particularly for the most lipophilic compounds. The observed differences between individual prediction methods are expected, because logP algorithms differ in their treatment of atom fragments, correction factors, solvation terms and training-set coverage. Physics-based, fragment-based, and atom-contribution approaches can therefore produce different estimates for the same molecule, especially when compounds contain fused aromatic systems, tertiary amines, ether linkers, carbonyl functions, or -CN substituents. Given the small set of six investigated compounds, the Pearson correlation coefficients and error metrics are used only as descriptive within-series indicators (Figure 2). They are not sufficiently robust for formal statistical ranking or for general conclusions about prediction-method performance across broader chemical space [41,69,70,71].

**Figure 2:**
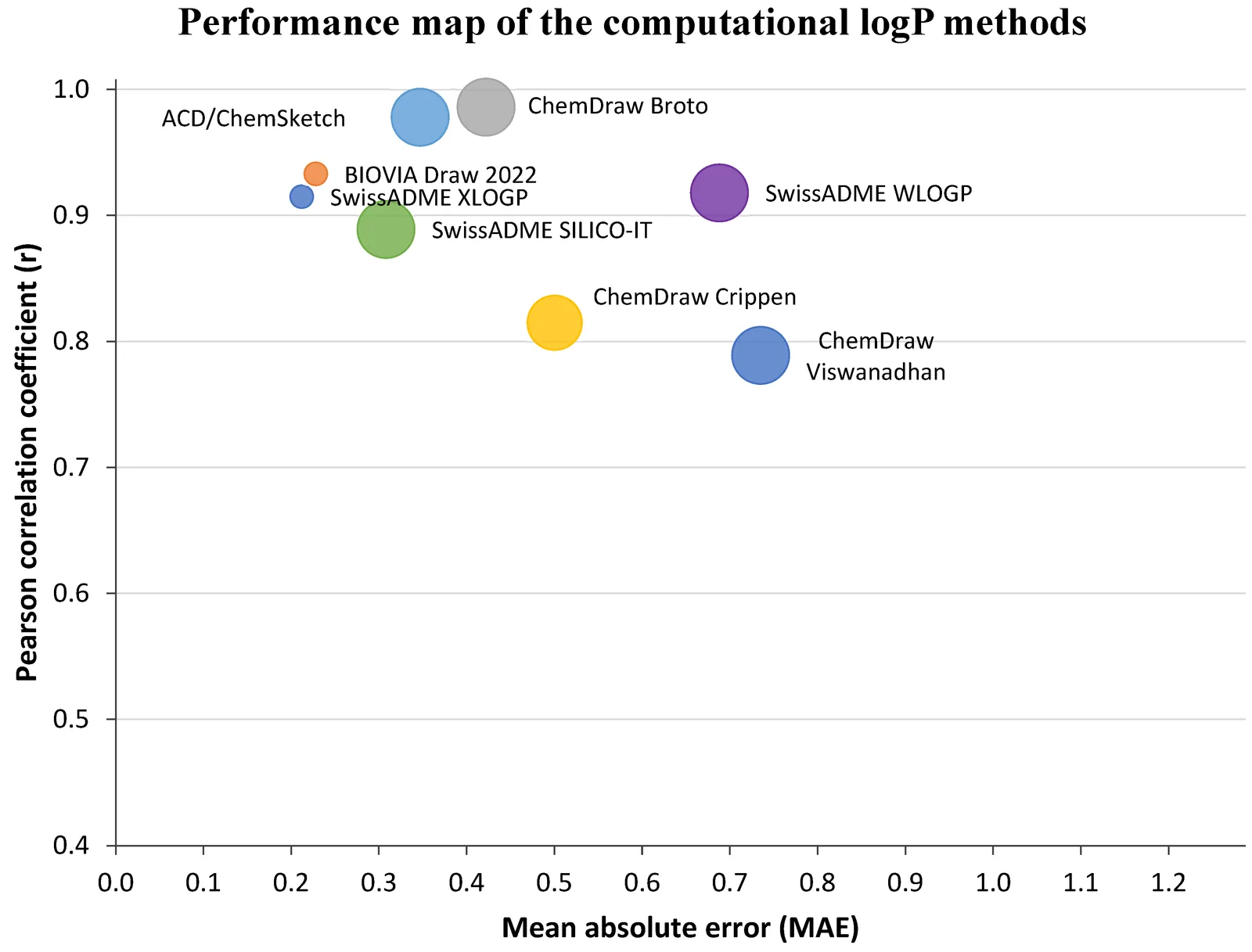
Descriptive comparison of in silico logP prediction methods against the HPLC-derived chromatographic lipophilicity estimates obtained at pH 7.4. The x-axis represents mean absolute error (MAE) and the y-axis Pearson correlation coefficient (r). Because only six compounds were investigated, these metrics are presented solely as exploratory descriptors for this series and are not used to establish a general performance ranking of the prediction methods.

### 3.3. Phospholipid Binding Analysis by Immobilized Artificial Membrane (IAM) Chromatography

The phospholipid binding of the newly developed putative anti-Alzheimer’s compounds was evaluated using an IAM column under physiological conditions (pH 7.4). The calibration set (Table 5) was chosen to cover a wide range of CHI_IAM_ values, thereby ensuring adequate representation across the full scale from low to high phospholipid affinity. To obtain the CHI_IAM_ values, linear regression analysis was performed between φ_0_ and the mean retention time from three consecutive gradient injections [26,72].

A strong linear relationship between gradient retention times and CHI_IAM_ values was established for the standard compounds on the IAM column and is described by the following equation:

(25) CHI_IAM_ = 18.13 × t_R_ − 69.67 *n* = 8; R^2^ = 0.99

The IAM chromatographic data (Table 6) showed that the investigated biphenylalkoxyamine-related H_3_ receptor ligands possess a high affinity for the phospholipid-mimetic stationary phase. The experimentally obtained CHI_IAM_ values for (**1**)–(**6**) ranged from 63.0 to 71.9, indicating a rather pronounced interaction with the membrane throughout the entire set of the compounds. In comparison, donepezil and rivastigmine exhibited substantially lower CHI_IAM_ values of 45.0 and 39.0, suggesting a weaker affinity to the phospholipids than the investigated derivatives.

Among the tested compounds, (**1**) showed the highest CHI_IAM_ value (71.9), closely followed by (**5**) (71.6), whereas (**6**) displayed the lowest value (63.0). The generally high CHI_IAM_ values observed for compounds (**1**)–(**6**) are consistent with their predominantly aromatic and lipophilic character in combination with the presence of a basic amino moiety, both of which are expected to exhibit a high interaction with the phospholipid-mimetic IAM phase [31,44,73]. Overall, only moderate differences were observed within the set of the compounds, indicating that all derivatives possess a similarly strong tendency for membrane interaction. At the same time, the somewhat lower value obtained for (**6**) may be related to the presence of the nitrile substituent, which increases the polarity of the molecule and may reduce the strength of hydrophobic interactions with the IAM phase, whereas the higher CHI_IAM_ values of compounds (**1**) and (**5**) suggest that their less polar and more extended hydrophobic aromatic frameworks are more favorable for interaction with the phospholipid-like stationary phase [45,74].

### 3.4. Plasma Protein Binding (PPB) Analysis

The plasma protein binding of the structurally related alkoxyamine H_3_ receptor ligands was assessed under physiological conditions (pH 7.4) using biomimetic HSA and AGP columns. Calibration results for the HSA column are presented in Table 7. The reference compounds were selected to cover a broad range of HSA-binding percentages, from 19% for cimetidine to 99.8% for diclofenac. A strong linear relationship was observed between gradient retention times and logk values (R^2^ = 0.96), correlating with previous reports [32]. The gradient elution retention times were then converted to logk according to Equation (26).

(26) $$logk=2.45*{logt}_{r}-0.13$$

Calibration of the AGP column was carried out using the standards presented in Table 8. The selected AGP-binding standards demonstrated a strong linear correlation (R^2^ = 0.96) between logk values and gradient retention times, allowing the same screening strategy to be applied to a protein phase that is especially relevant for basic and cationic drugs [75]. Retention times measured on the AGP column were converted to logk values with the use of Equation (27).

(27) $$logk=2.71\times {logt}_{r}-0.99$$

Overall, the calibration of both the HSA and AGP biomimetic columns showed strong linear relationships between gradient retention times and log*k* values, confirming the reliability of the applied approach for a fast screening.

The biomimetic plasma protein binding data showed that all synthesized structurally related alkoxyamine H_3_ receptor ligands exhibited high affinity towards both HSA and AGP biomimetic columns (Table 9). For the HSA column, the tested compounds exhibited logk values in a range between 1.42–1.55, corresponding to narrow percentage difference (97.3–98.2%) in the predicted plasma protein binding. This indicates consistently strong interaction with the human serum albumin protein. The affinity to the AGP was also high (>80%), although slightly lower, with log*k* values of 0.67–0.85 and predicted binding values of 83.2–88.6% [76]. This trend suggests that the compounds have a generally greater affinity for albumin than for α1-acid glycoprotein.

The relatively high protein binding observed for compounds (**1**)–(**6**) can likely be attributed to their lipophilic character, resulting from the presence of aromatic systems in combination with a basic amino moiety. The limited variation in HSA binding among the derivatives indicates that structural modifications within this series had only a minor effect on the affinity to albumin [75,77,78]. In contrast, slightly larger differences were observed for AGP, suggesting that AGP-based interactions may be somewhat more susceptible to subtle structural changes. Among the tested compounds, (**1**) showed the highest predicted HSA binding (98.2%), whereas (**3**) the highest AGP binding (88.6%). (**6**) showed the lowest AGP affinity (83.2%), which may reflect the influence of the nitrile substituent and is in line with its lower pH 7.4 HPLC-derived chromatographic lipophilicity estimate and lower CHI_IAM_ value.

The experimentally determined PPB values for donepezil and rivastigmine were highly consistent with previously reported literature data, supporting the reliability of the applied analytical method. Donepezil showed a PPB value of 94.6%, which is close to the reported value of approximately 96% [79]. This confirms its extensive association with plasma proteins, a property that may affect its pharmacokinetic behavior and tissue distribution. By contrast, rivastigmine displayed markedly lower plasma protein binding, with an experimental PPB of 39.8%, in agreement with literature values of around 40% [11].

When compared with the reference standards, all tested derivatives showed noticeably higher plasma protein binding behavior than rivastigmine, while the differences relative to donepezil were much less pronounced. The binding profile of the compounds was generally comparable to that of donepezil. Donepezil also exhibited high binding on both biomimetic columns, with values only slightly lower than those observed for compounds (**1**)–(**6**), whereas rivastigmine showed weaker interactions, particularly on the AGP column. Overall, these results indicate that the synthesized derivatives have a strong tendency to bind plasma proteins.

The biomimetic plasma protein binding data correlated with the IAM results, as both assays indicated a strong interaction tendency of compounds (**1**)–(**6**) with hydrophobic biological environments. All derivatives showed very high HSA binding and high AGP binding, which corresponded well with their high CHI_IAM_ values and thus pronounced affinity for the phospholipid-mimetic stationary phase. (**1**) and (**5**) belonged to the most strongly PPB-bound compounds and exhibited the highest CHI_IAM_ values, whereas compound (**6**) showed the lowest AGP-related binding and the lowest CHI_IAM_ value within the series. Overall, the combined PPB and IAM findings indicate that the derivatives are characterized by extensive plasma protein binding together with strong membrane affinity, which may influence their distribution-related pharmacokinetic properties. However, high plasma protein binding alone should not be taken as a direct indicator of longer systemic exposure or prolonged pharmacological effect, since its pharmacokinetic impact is influenced by factors such as unbound intrinsic clearance, tissue distribution, extraction ratio, and variations in plasma protein levels [80,81].

### 3.5. Biomimetic Distribution Modelling

The predicted distribution data (Table 10) suggest that the investigated structurally related alkoxyamine H_3_ receptor ligands may have a pronounced tendency for tissue distribution. Compounds (**1**)–(**6**) showed logV_d_ values ranging from 2.29 to 3.98, which corresponds to Vd values between 198.8 and 9619.2 L/kg, indicating extensive tissue distribution [34]. Within the series, the highest Vd values were observed for compounds (**1**) and (**5**), whereas (**6**) showed the lowest value. Compounds (**2**)–(**4**) displayed intermediate behavior. This pattern appears to be in agreement with previously mentioned IAM data, as compounds (**1**) and (**5**) also showed the highest CHI_IAM_ values, whereas (**6**) exhibited the lowest phospholipid affinity [82]. A model-derived V_d_ in the range of several thousand L/kg is far beyond the magnitude typically encountered for marketed drugs and signals substantial extrapolation of the biomimetic relationship rather than a literal physiological volume [83]. A very large predicted volume of distribution should not be interpreted as intrinsically advantageous, because extensive tissue partitioning can coexist with a low unbound fraction and does not by itself establish adequate pharmacologically active brain exposure [84]. In comparison, the corrected biomimetic calculation for donepezil gives a lower model-derived V_d_ of 3.27 L/kg. This model estimate should not be expected to reproduce the reported clinical values of 11.8 ± 1.70 L/kg after 5 mg and 11.6 ± 1.91 L/kg after 10 mg; the clinical values are included only as contextual observations rather than as a direct validation of the model [79]. Rivastigmine (model-derived V_d_ = 4.70 L/kg) displayed a more moderate distribution profile, likely due to its lower lipophilicity and weaker plasma protein binding, consistent with reported literature values ranging from 2.8 to 5.5 L/kg [85]. Clinical pharmacokinetic-pharmacodynamic modelling has also described rivastigmine as a compound with rapid systemic disposition and a more limited distribution profile than highly lipophilic CNS drugs [85].

Additionally, all tested compounds showed a very high predicted brain tissue binding (%BTB = 97.1–97.6%), suggesting a strong tendency to associate with brain tissue components. This is particularly relevant for CNS-active compounds, as brain tissue binding may influence both the extent of total brain distribution and the unbound drug fraction available for pharmacological efficacy [86]. Accordingly, the high %BTB values observed here suggest strong brain tissue association, but they may also imply a lower unbound fraction within brain tissue. This distinction is important because modern CNS pharmacokinetic assessment increasingly emphasizes unbound brain-to-plasma exposure relationships, such as K_p, uu, brain_ rather than total brain concentrations alone [87].

Taken together, the high model-derived V_d_ and %BTB values suggest that the synthesized derivatives could combine extensive tissue distribution with high tendency for brain tissue binding. A similar trend was observed for brain tissue binding, where donepezil (%BTB = 95.3) remained slightly below the investigated derivatives, while rivastigmine (%BTB = 74.5) showed substantially weaker binding. Overall, these model outputs indicate a greater predicted tendency for tissue distribution and brain-tissue association than the two reference drugs, but they do not constitute direct evidence of in vivo distribution.

The HSA/IAM-derived V_d_, V_du_, and BTB outputs should therefore be regarded as comparative model-based descriptors rather than direct measurements of in vivo pharmacokinetics. The exceptionally large V_d_ estimates for compounds (**1**) and (**5**) arise from extrapolation of the empirical HSA/IAM relationships into a highly lipophilic, strongly membrane-binding region and should not be interpreted literally as clinical distribution volumes. Accordingly, relative ranking is more defensible than the absolute magnitude of these extreme estimates. These model outputs, together with the IAM-based BBB and HIA estimates, require confirmation using direct unbound-fraction, tissue-exposure, transporter, and in vivo pharmacokinetic measurements.

### 3.6. Plasma Stability

The first-order analysis of the seven mean time-point responses for the 12 compound–matrix profiles (Supplementary Information (SI) table (Tables S1–S12) is summarized in Table 11. Human plasma retained 77.5–89.5% of parent compound after 24 h, whereas rat plasma retained 62.2–80.5%. Apparent half-lives ranged from 71 to 228 h in human plasma and from 42 to 122 h in rat plasma. Importantly, all 12 apparent half-lives exceeded the 24 h experimental window and therefore required extrapolation beyond the observed period. The goodness of fit varied across profiles (R^2^ = 0.414–0.918), reflecting the limited decline observed for several highly stable compounds.

The 95% confidence intervals further illustrate the uncertainty associated with the longest estimates. For compounds (**3**) in rat plasma, (**4**) in rat plasma, (**5**) in rat plasma and (**6**) in human plasma, the 95% confidence interval of *k*_obs_ included zero; consequently, the upper confidence limit of t_1/2_ was unbounded and only a lower half-life confidence bound could be reported. Among profiles with bounded confidence intervals, compound (**1**) showed an apparent t_1/2_ of 166 h (95% CI: 105–394 h) in human plasma and 42 h (23–310 h) in rat plasma, whereas compound (**6**) showed 87 h (47–582 h) in rat plasma. These values are most appropriate for comparative ranking of plasma stability and should not be interpreted as precise predictions of in vivo persistence.

Compound (**1**), for example, showed a half-life of 166 h in human plasma but only 42 h in rat plasma. Similarly, (**2**), (**3**), and (**4**) were all less stable in rat plasma than in human plasma. By contrast, compound (**5**) showed slightly higher parent-compound recovery in rat than in human plasma after 24 h, whereas (**6**) remained somewhat less stable in rat than in human plasma; these interspecies differences were smaller than those observed for compounds (**1**) and (**2**) [88,89]. With regard to their chemical structure, compounds (**1**)–(**4**) are closely related, consisting of cyclic tertiary amino moiety connected through an alkoxyalkyl linker to a hydrophobic biaryl-type aromatic system. Thus, their plasma stability profiles were largely similar, especially in human plasma, where only moderate differences in half-life were observed. This suggests that changes in the arrangement of the distal aromatic ring system within this subgroup had only a slight influence on stability in human plasma. In rat plasma, however, (**1**)–(**4**) showed a broader difference of half-life values, indicating that subtle differences in aromatic topology may influence susceptibility to degradation more strongly under enzymatic activity of the rat plasma. Since these compounds all contain the same basic aminoalkyl-ether linkage, the observed differences are likely related not to the presence or absence of a hydrolyzable functional group, but rather to changes in steric environment and/or electronic distribution around the aromatic portion of the molecules.

(**5**) differs more substantially from (**1**)–(**4**), as it contains a fused tricyclic aromatic scaffold with a carbonyl-containing xanthone-like core. This difference in comparison to the other compounds may reduce conformational freedom and potentially limit access of plasma enzymes to the alkoxy side chain, which could contribute to its relatively favorable stability, particularly seen in rat plasma. Although (**5**) was the least stable derivative in human plasma, it showed the highest stability in rat plasma, suggesting that its more condensed aromatic system may be especially advantageous under conditions where degradation is otherwise more pronounced. This behavior distinguishes (**5**) from the other derivatives and indicates that increased scaffold rigidity may be beneficial for plasma stability in rat species.

Compound (**6**) also differs from the other derivatives by having a nitrile substituent on the aromatic ring and by possessing a shorter alkoxy linker between the cyclic amine and the aromatic moiety. Between all the compounds in the set, it showed the highest stability in human plasma and the second highest stability in rat plasma. This may be associated with the combined influence of the polar nitrile substituent and the somewhat more compact molecular arrangement in comparison to other derivatives. The nitrile group in the compound (**6**) may alter the nature of the aromatic system and reduce the overall susceptibility of the molecule to degradation, while the shorter linker may decrease conformational flexibility and thereby limit interactions with plasma enzymes. Summarized, (**6**) appears to combine high plasma stability with a structural motif that is less accessible to enzymatic activity than the other analogues.

Compared with the reference drugs donepezil and rivastigmine, the analogues appear to exhibit a plasma stability profile more closely resembling donepezil than rivastigmine. Donepezil is characterized by a relatively long half-life, commonly reported to be around 79.5 ± 19.0 h, whereas rivastigmine undergoes much more rapid clearance, with a plasma half-life of about 1 h, consistent with its susceptibility to hydrolysis [12,90].

The clinical elimination half-lives of donepezil and rivastigmine are not directly comparable with the apparent in vitro plasma-stability half-lives reported here, as in vivo half-life also reflects distribution, hepatic and extrahepatic metabolism, renal elimination, and other clearance processes [25,89,90].

Overall, the parent-compound recovery and first-order regression parameters support generally greater plasma stability in human than in rat plasma for most of the series, while also showing compound-specific exceptions. The species difference is compatible with known differences in plasma hydrolase composition and activity [88,89,91]. Because degradation was limited over the 24 h observation period, the plasma-stability experiment is most informative for comparative ranking within this series. It does not establish systemic clearance, duration of action, dosing frequency, accumulation risk or clinical half-life, which require additional metabolic and in vivo pharmacokinetic measurements [25,92,93].

### 3.7. Solubility

The solubility data (Table 12) showed that all investigated compounds exhibited moderate to relatively high aqueous solubility across the three tested media. Values ranged from 123.5 to 170.8 µM in the acidic gastric-simulating JP1 medium (pH 1.2), from 118.8 to 169.9 µM in JP2 (pH 6.8) and from 53.2 to 164.9 µM in phosphate buffer at pH 7.4. The measured CV values were low in all cases (0.2–4.7%), indicating good assay reproducibility. As the assay was performed after a 1 h incubation following dilution from DMSO stock solutions, these values are most appropriately regarded as operational screening-solubility estimates rather than equilibrium thermodynamic solubilities, which is consistent with the intended early-stage use of this method [35]. Since solubility is closely related to absorption, formulation and overall developability, these results are relevant for assessing the pharmaceutical potential of the synthesized derivatives [94,95,96].

At pH 1.2, (**5**) showed the highest solubility (170.8 µM), followed by (**3**) (157.5 µM), (**2**) (156.2 µM), (**1**) (149.0 µM) and (**4**) (148.6 µM), whereas (**6**) showed the lowest JP1 value (123.5 µM). In JP2, (**5**) again had the highest solubility (169.9 µM), while (**6**) had the lowest (118.8 µM). At pH 7.4, the ranking changed, with (**2**) being the most soluble (164.9 µM) and (**1**) the least soluble (53.2 µM). (**1**) therefore showed the largest decrease between JP1 and pH 7.4, whereas (**2**) maintained high solubility across all three media and was slightly more soluble at pH 7.4 than in JP1. The absence of a strictly monotonic pH trend for every compound indicates that ionization is important but does not alone determine the measured screening solubility.

The tested compounds were evaluated in their oxalate salt forms, consistent with the chemical form used throughout the experimental workflow. Salt formation can enhance aqueous solubility relative to the corresponding free base, but the magnitude of the effect depends on the ionization state, counterion, medium composition, and solid-state behavior [95,96]. The pronounced aromatic and lipophilic character of the compounds would generally oppose aqueous dissolution. However, all six structures contain a protonatable tertiary cyclic amine, which would be expected to be extensively protonated in JP1 at pH 1.2. The resulting increase in ionic hydration provides a plausible explanation for the relatively high JP1 solubility of the complete series despite their hydrophobic aromatic domains. The data also shows that the oxalate-salt measurements should not be extrapolated directly to the corresponding free bases [97,98,99].

Among the closely related biphenyl azepane derivatives, compounds (**1**), (**3**) and (**4**) showed a relatively narrow JP1 range (148.6–157.5 µM), suggesting that strong protonation at pH 1.2 reduces the influence of positional biphenyl substitution on the observed solubility. (**5**) with its xanthone scaffold, showed the highest JP1 value despite its extended aromatic surface; its additional carbonyl and ether oxygen atoms may contribute to hydration of the protonated molecule. In contrast, (**6**), the cyanobiphenyl analogue, had the lowest JP1 solubility despite possessing the lowest pH 7.4 chromatographic lipophilicity in the series. The nitrile increases polarity but is not itself appreciably ionized under these conditions, and the result illustrates that solubility cannot be predicted from lipophilicity or a single polar substituent alone; crystal packing, salt behavior, and other solid-state factors may also contribute [74,94].

Overall, the three-medium profile supports acceptable screening solubility for early-stage development, while the pronounced pH sensitivity of (**1**) and the compound-specific deviations from a simple ionization trend justify subsequent thermodynamic-solubility, salt-screening and solid-state studies.

### 3.8. Integrated ADME and Biological Lead Prioritization

To provide a single within-series prioritization, the experimental and biomimetic ADME results were integrated with the biological activities reported previously for the same analogues (Table 1). The biological endpoints were not re-measured in the present study and because the pharmacological dataset contains heterogeneous readouts (K_i_ values, IC_50_ values, % inhibition at fixed concentrations and in vivo observations), no separate numerical biological score was constructed, since this would imply a degree of quantitative comparability that the source data does not support. Instead, the final ranking is an evidence-based synthesis of ADME balance and reported pharmacology and is intended only for hypothesis-generating prioritization within this six-compound series.

The prioritization deliberately avoided treating every higher numerical value as favorable. Plasma stability was judged primarily from the directly observed percentage of parent compound remaining at 24 h rather than from extrapolated half-lives. Very high PPB was considered a potential limitation rather than an advantage and P_m_, HIA, V_d_, V_du_ and BTB were used as comparative biomimetic descriptors rather than direct in vivo measurements. Lower-to-moderate lipophilicity within this highly lipophilic series was favored and solubility was considered across all three media. The JP1 measurement was used specifically to assess behavior under strongly acidic gastric-like conditions and the extent to which solubility changed across pH. For the biological component, the H_3_R affinity was treated as the primary target criterion with BuChE/AChE/MAO-B activity and reported in vivo CNS or memory-related evidence providing additional support. A ‘not tested’ result was treated as absence of evidence rather than evidence of inactivity.

The integrated ranking placed (**6**) first (Table 13). It combines the strongest reported H_3_R affinity in the series (K_i_ = 9.2 nM) with low-micromolar AChE, BuChE, and MAO-B inhibition and a beneficial effect in the scopolamine-induced memory-impairment model. Its ADME profile is also comparatively balanced: 89.5% and 79.9% parent compound remained after 24 h in human and rat plasma, respectively. It had the lowest pH 7.4 HPLC-derived lipophilicity estimate (4.81), the least extreme model-derived V_d_ (198.8 L/kg), and maintained solubility of 123.5/118.8/110.4 µM in JP1/JP2/pH 7.4. Although its JP1 solubility was the lowest within the six-compound set, the value remained above 120 µM and showed no marked collapse at near-neutral pH. (**5**) ranked second because its broader pharmacological profile—potent AChE and MAO-B inhibition, submicromolar BuChE inhibition and reported memory-enhancing activity—was coupled with the best acidic and intestinal solubility (170.8/169.9 µM in JP1/JP2) and favorable rat-plasma stability. Its high lipophilicity and extremely large model-derived V_d_ remain its biggest liabilities. (**2**) ranked third: it combined good H_3_R affinity and consistently high solubility across JP1, JP2, and pH 7.4, but its cholinesterase and MAO-B activities were not tested and its rat-plasma stability was lower than that of the two leading compounds. (**3**) ranked fourth because it combined the most potent reported BuChE inhibition in the series (IC50 = 0.447 µM) with moderate H_3_R affinity and good three-medium solubility, but it lacked in vivo evidence and retained high lipophilicity and protein binding. (**4**) ranked fifth: its ADME profile was relatively balanced, including 80.8/75.4% parent remaining in human/rat plasma and solubility of 148.6/138.5/105.3 µM, but it had the weakest H_3_R affinity (K_i_ = 528 nM), which lowered its integrated priority despite submicromolar BuChE inhibition. Compound (**1**) ranked sixth. Although it showed submicromolar BuChE inhibition and good human-plasma stability, it combined the highest pH 7.4 chromatographic lipophilicity (6.55), very high PPB, an extreme model-derived V_d_, the lowest rat-plasma stability (62.2% remaining at 24 h), and the strongest pH-dependent solubility loss (149.0 µM in JP1 versus 53.2 µM at pH 7.4). The final order, (**6**) > (**5**) > (**2**) > (**3**) > (**4**) > (**1**), should therefore be viewed as a transparent within-series synthesis of available evidence rather than a validated predictive model.

**Table 13:** Integrated ADME summary and final within-series prioritization of compounds (**1**)–(**6**). Values are shown as human/rat parent compound remaining at 24 h, HSA/AGP binding, CHI_IAM_/P_m_/%HIA, Vd/log Vdu/BTB, pH 7.4 HPLC-derived chromatographic lipophilicity and JP1/JP2/pH 7.4 solubility. The final rank additionally incorporates the previously reported H_3_R, cholinesterase/MAO-B, and in vivo evidence summarized in Table 1. All six compounds were classified as CNS-positive by the IAM model.

Compound	Human/Rat Plasma 24 h Remaining (%)	HSA/AGP PPB (%)	CHI_IAM_/P_m_/%HIA	Vd/log Vdu/BTB	HPLC logP (pH 7.4)	Solubility JP1/JP2/pH 7.4 (µM)	Rank
(6)	89.5/79.9	97.3/83.2	63.0/24.7/80.9	198.8/3.98/97.1	4.81	123.5/118.8/110.4	**1**
(5)	77.5/80.5	97.4/87.8	71.6/21.9/83.4	9619.2/5.65/97.5	5.73	170.8/169.9/123.9	**2**
(2)	81.4/69.3	97.3/87.9	65.7/42.4/82.1	579.6/4.43/97.2	5.56	156.2/143.4/164.9	**3**
(3)	78.2/70.7	97.9/88.6	66.7/14.9/83.3	736.4/4.70/97.4	6.16	157.5/142.5/127.8	**4**
(4)	80.8/75.4	97.7/88.0	64.6/19.0/82.3	327.6/4.30/97.2	6.27	148.6/138.5/105.3	**5**
(1)	89.0/62.2	98.2/88.1	71.9/23.4/85.0	9224.1/5.85/97.6	6.55	149.0/128.0/53.2	**6**

## 4. Conclusions

The present study establishes a comparative biomimetic ADME profile for six multifunctional biphenylalkoxyamine-related histamine H_3_ receptor ligands and demonstrates that biological activity should be evaluated together with physicochemical and pharmacokinetic-related developability. Reanalysis of the plasma-stability data using first-order regression across seven time points showed limited parent-compound loss over 24 h in both human and rat plasma. All apparent half-lives extended beyond the experimental window, and several estimates had broad or unbounded confidence intervals; these values are therefore comparative in vitro stability descriptors rather than predictors of clinical elimination half-life, systemic exposure or dosing frequency.

Plasma protein binding was high throughout the series and should be regarded as a balance parameter rather than an intrinsic advantage. The IAM- and HSA-derived P_m_, HIA, V_d_, V_du_, and BTB outputs provide useful comparative descriptors but are biomimetic predictions rather than direct in vivo pharmacokinetic measurements. The series was highly lipophilic by the pH 7.4 HPLC method, yet the oxalate salts remained measurably soluble across the three tested media. JP1 solubility at pH 1.2 ranged from 123.5 to 170.8 µM and was generally higher than or comparable with the near-neutral values, consistent with extensive protonation of the tertiary amines under acidic conditions. The similar JP1 values of the biphenyl positional isomers (**1**), (**3**) and (**4**), the highest JP1 solubility of the xanthone derivative (**5**) and the comparatively lower value of the nitrile-containing analogue (**6**) show that ionization is a major contributor but that aromatic topology, additional polar groups, salt form and solid-state behavior also shape the observed solubility. The pronounced fall for (**1**) from 149.0 µM in JP1 to 53.2 µM at pH 7.4 further emphasizes the need to characterize pH-dependent solubility rather than relying on a single medium.

When the ADME profile is considered together with the previously reported pharmacological evidence in one integrated ranking, the final order is (**6**) > (**5**) > (**2**) > (**3**) > (**4**) > (**1**). Compound (**6**) is the principal potency–developability lead due to its combination of the strongest H_3_R affinity, reported memory-related in vivo activity, favorable cross-species plasma stability, the lowest chromatographic lipophilicity in the series, the least extreme model-derived distribution and stable solubility across the three media. (**5**) is the pharmacology-oriented co-lead, combining potent AChE/BuChE/MAO-B activity and reported in vivo efficacy with the highest JP1 and JP2 solubility, although its high lipophilicity and extreme model-derived Vd remain liabilities. (**2**) ranks third because of good H_3_R affinity and consistently high solubility, but the absence of cholinesterase/MAO-B data limits the strength of its integrated evidence. Future work should prioritize direct pH-dependent logD measurements, thermodynamic solubility and solid-state/salt characterization, unbound plasma and brain fractions, metabolic stability, transporter and efflux assessment and confirmatory in vivo pharmacokinetic and efficacy studies. The integrated ranking is exploratory and should be validated in broader chemical series.

## Figures and Tables

**Figure 1:**
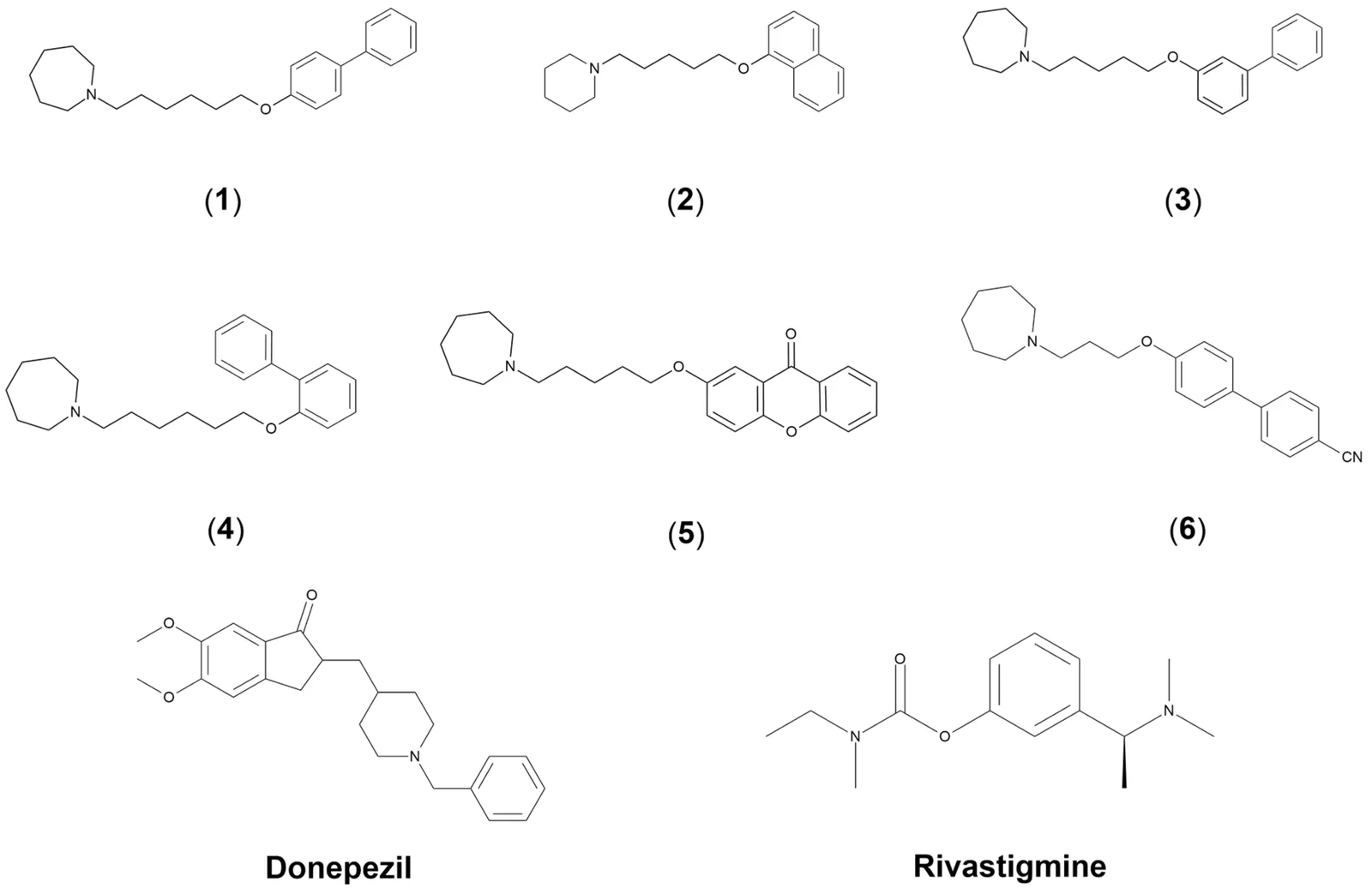
Series of investigated biphenylalkoxyamine derivatives and structurally related analogues (**1**–**6**).

**Table 1:** Reported biological activities of compounds (**1**)–(**6**). The table summarizes *h*H_3_R affinity, cholinesterase and MAO activity, and additional receptor or in vivo findings. Activity is reported as K_i_, IC_50_, or percentage inhibition at the indicated concentration.

Compound	*h*H_3_R Affinity K_i_ (nM)	Cholinesterase Activity	*h*MAO-B Activity	Additional Receptor/ In Vivo Findings
(**1**)	150.9 ± 72.8	*ee*AChE: IC_50_ = 2.76 ± 0.04 µM *eq*BuChE: IC_50_ = 0.599 ± 0.010 µM	45 ± 1% inhibition at 1 µM	Not evaluated in vivo.
(**2**)	53.9 [28.0–104]	n.t. *	n.t. *	hH_1_R K_i_ = 1.82 ± 0.20 µM; hH_4_R K_i_ = 58.5 ± 6.6 µM. Anticonvulsant activity in MES and 6 Hz seizure models [37].
(**3**)	114.7 ± 22.0	*ee*AChE: 26 ± 3% inhibition at 10 µM *eq*BuChE: IC_50_ = 0.447 ± 0.006 µM	14 ± 0% inhibition at 1 µM	Not evaluated in vivo.
(**4**)	528 [328–849]	*ee*AChE: 30 ± 5% inhibition at 10 µM *eq*BuChE: IC_50_ = 0.686 ± 0.045 µM	17 ± 0% inhibition at 1 µM	Not evaluated in vivo.
(**5**)	170 ± 10	*ee*AChE: IC_50_ = 0.180 ± 0.003 µM *eq*BuChE: IC_50_ = 0.880 ± 0.026 µM	IC_50_ = 0.775 ± 0.111 µM	Memory-enhancing activity in dizocilpine-induced amnesia; analgesic effects in neuropathic pain models.
(**6**)	9.2 [1.0–83]	*ee*AChE: IC_50_ = 2.638 ± 0.048 µM *eq*BuChE: IC_50_ = 1.299 ± 0.010 µM	IC_50_ = 1.407 ± 0.123 µM	Beneficial effect in a scopolamine-induced memory-impairment model.

* n.t., not tested.

**Table 2:** Biomimetic blood–brain barrier permeability estimation based on immobilized artificial membrane chromatography. MW—molecular weight; P_m_—coefficient of membrane permeability; k_IAM_—retention factor on the IAM biomimetic column; CV—coefficient of variation calculated from three separate measurements.

Compound	CNS	MW	P_m_	*k*_IAM_ [*n* = 3] ± CV [%]
(**1**)	+	351.5	23.4	35.73 ± 0.61
(**2**)	+	297.4	42.4	33.21 ± 0.76
(**3**)	+	337.5	14.9	19.37 ± 0.53
(**4**)	+	351.5	19.0	28.99 ± 3.62
(**5**)	+	379.5	21.9	45.32 ± 0.25
(**6**)	+	334.5	24.7	30.90 ± 0.19
Donepezil	+	416.0	2.20	6.60 ± 0.60
Rivastigmine	+	250.3	4.46	1.75 ± 0.15

**Table 3:** Human intestinal absorption estimation based on IAM chromatography. %HIA—percentage of human intestinal absorption; PSA—polar surface area; logP—logarithm of the partition coefficient; *k*_IAM_—retention factor on the IAM biomimetic column. The logP column is the calculated descriptor required by the published QSAR and is distinct from the HPLC-derived chromatographic lipophilicity estimate measured at pH 7.4.

Compound	%HIA	PSA	logP	PSA × logP	1/k_IAM_ × logP	1/k_IAM_	k_IAM_
(**1**)	85.0	12.47	6.40	79.79	0.59	0.09	10.91
(**2**)	82.1	12.47	4.88	60.81	1.51	0.31	3.22
(**3**)	83.3	12.47	5.94	74.11	0.95	0.16	6.28
(**4**)	82.3	12.47	6.40	79.79	0.86	0.13	7.43
(**5**)	83.4	38.77	5.56	215.51	1.35	0.24	4.10
(**6**)	80.9	36.26	4.79	173.52	1.73	0.36	2.77
Donepezil	85.8	38.77	4.57	177.17	1.28	0.28	3.52
Rivastigmine	68.7	32.78	2.60	85.21	3.46	1.33	0.75

**Table 4:** HPLC-derived chromatographic lipophilicity estimates at pH 7.4 and in silico calculated logP values of the investigated alkoxyamine H_3_ receptor ligands and reference drugs. The HPLC-derived values are reported under the experimental logP convention of the calibration method but should be interpreted as pH 7.4 chromatographic lipophilicity estimates; SD—standard deviation based on three measurements; SwissADME, BIOVIA Draw, ChemDraw and ACD/ChemSketch—in silico logP estimates.

Compound	HPLC logP ± SD	SwissADME					BIOVIA Draw 2022	ChemDraw 23			ACD/ChemSketch 2021.2.1
		iLOGP	XLOGP	WLOGP	MLOGP	SILICO-IT		Broto	Viswanadhan	Crippen	
(**1**)	6.55 ± 0.069	4.74	6.19	5.79	4.60	6.13	6.40	6.18	5.87	6.13	7.05
(**2**)	5.56 ± 0.027	4.04	5.89	4.49	3.78	4.93	4.88	4.92	4.40	4.61	5.53
(**3**)	6.16 ± 0.021	4.47	5.83	5.40	4.39	5.74	5.94	5.72	5.48	5.71	6.47
(**4**)	6.27 ± 0.016	4.58	6.19	5.79	4.60	6.13	6.40	6.18	5.87	6.13	6.90
(**5**)	5.73 ± 0.010	4.57	5.88	4.99	3.17	5.67	5.56	5.24	4.39	4.75	6.04
(**6**)	4.81 ± 0.003	4.01	4.83	4.49	3.22	4.99	4.79	4.31	4.66	4.87	5.11
Donepezil	3.93 ± 0.001	3.92	4.28	3.83	3.07	4.00	4.57	3.65	3.69	4.01	4.71
Rivastigmine	2.14 ± 0.002	3.21	2.29	2.44	2.34	1.46	2.60	Ø	2.41	2.36	2.14

**Table 5:** Set of compounds used for calibration of CHI on IAM column at physiological pH (7.4). t_R_—retention time; S—slope calculated based on 5 different acetonitrile concentrations; log*k*_w_—intercept calculated based on 5 different acetonitrile concentrations; CHI_IAM_—chromatographic hydrophobicity index on IAM biomimetic column.

Compound	t_R_ ± SD	S	log*k*_w_	CHI_IAM_
Paracetamol	4.093 ± 0.004	−0.026	−0.130	4.9
Chlorothiazide	4.154 ± 0.001	−0.046	0.253	5.5
Propyphenazone	5.365 ± 0.005	−0.041	1.041	25.4
Propil *p*-hydroxybenzoate	5.855 ± 0.004	−0.049	1.808	36.7
Valerophenone	5.945 ± 0.002	−0.049	1.892	38.7
2,3-Dichlorophenol	6.148 ± 0.004	−0.045	1.875	41.5
Octanophenone	6.425 ± 0.004	−0.060	2.972	49.4
Tamoxifen	7.621 ± 0.004	−0.036	2.399	67.3

**Table 6:** CHI_IAM_ lipophilicity data of the structurally related alkoxyamine H_3_ receptor ligands. t_R_—retention time; SD—standard deviation based on three measurements; CHI_IAM_—chromatographic hydrophobicity index based on IAM chromatography; logK_IAM_—logarithm of IAM equilibrium partition coefficient; log*k*_IAM_—logarithm of IAM retention factor.

Compound	t_R_ ± SD	CHI_IAM_	logK_IAM_	log*k*_IAM_
(**1**)	7.811 ± 0.008	71.9	12.773	3.729
(**2**)	7.466 ± 0.006	65.7	9.757	3.441
(**3**)	7.525 ± 0.008	66.7	10.209	3.490
(**4**)	7.407 ± 0.003	64.6	9.318	3.392
(**5**)	7.790 ± 0.001	71.6	12.564	3.711
(**6**)	7.318 ± 0.007	63.0	8.701	3.317
Donepezil	6.323 ± 0.002	45.0	4.192	2.488
Rivastigmine	5.993 ± 0.015	39.0	3.351	2.213

**Table 7:** Set of compounds used for the calibration of the biomimetic HSA column. t_R_—retention time; logt_R_—logarithm of the retention time; log*k*—logarithm of the apparent affinity constant; %PPB—percentage binding to HSA.

Compound	t_R_ ± SD	logt_R_	log*k* (Measured)	log*k* (Literature)	%PPB (Literature)
Acetylsalicylic acid	0.923 ± 0.007	−0.035	−0.22	−0.03	49
Budesonide	3.075 ± 0.006	0.488	1.06	0.83	88
Cimetidine	0.761 ± 0.015	−0.119	−0.42	−0.64	19
Ciprofloxacin	0.973 ± 0.004	−0.012	−0.16	−0.18	40
Diazepam	3.910 ± 0.002	0.592	1.32	1.63	98.7
Diclofenac	6.087 ± 0.006	0.784	1.79	1.92	99.8
Indomethacin	6.575 ± 0.012	0.818	1.87	1.69	99
Ketoprofen	5.843 ± 0.102	0.767	1.74	1.69	99
Modafinil	1.625 ± 0.014	0.211	0.38	0.17	60
Nicardipine	4.036 ± 0.036	0.606	1.35	1.20	95
Nizatidine	0.732 ± 0.019	−0.136	−0.46	−0.28	35
Propranolol	2.337 ± 0.008	0.369	0.77	0.79	87
Warfarin	4.637 ± 0.014	0.666	1.50	1.69	99

**Table 8:** Set of compounds used for the calibration of the biomimetic AGP column. t_R_—retention time; logt_R_—logarithm of the retention time; log*k*—logarithm of the apparent affinity constant; %PPB—percentage binding to AGP.

Compound	t_R_ ± SD	logt_R_	log*k* (Measured)	log*k* (Literature)	%PPB (Literature)
Carbamazepine	3.359 ± 0.005	0.526	0.43	0.26	65
Indomethacin	3.160 ± 0.002	0.500	0.36	0.09	56
Nicardipine	3.989 ± 0.002	0.601	0.64	0.79	87
Nizatidine	1.880 ± 0.004	0.274	−0.25	−0.24	37.1
Paracetamol	0.637 ± 0.001	−0.196	−1.53	−1.49	3.2
Propranolol	3.907 ± 0.010	0.592	0.61	0.76	86
Warfarin	3.820 ± 0.004	0.582	0.59	0.67	83.2

**Table 9:** Biomimetic PPB data of the investigated compounds. t_R_—retention time; SD—standard deviation based on three separate measurements; logt_R_—logarithm of the retention time; log*k*—logarithm of the apparent affinity constant; %PPB—percentage of plasma protein binding.

Compound	t_R_ HSA ± SD	logt_R_ HSA	log*k*_HSA_	%PPB_HSA_	t_R_ AGP ± SD	logt_R_ AGP	log*k*_AGP_	%PPB_AGP_
(**1**)	4.869 ± 0.007	0.687	1.55	98.2	4.725 ± 0.008	0.674	0.84	88.1
(**2**)	4.423 ± 0.010	0.635	1.42	97.3	4.683 ± 0.004	0.671	0.83	87.9
(**3**)	4.665± 0.011	0.669	1.50	97.9	4.791 ± 0.007	0.680	0.85	88.6
(**4**)	4.556 ± 0.017	0.659	1.48	97.7	4.706 ± 0.005	0.673	0.83	88.0
(**5**)	4.380 ± 0.009	0.641	1.44	97.4	4.674 ± 0.008	0.670	0.82	87.8
(**6**)	4.332 ± 0.011	0.637	1.42	97.3	4.109 ± 0.011	0.614	0.67	83.2
Donepezil	3.418 ± 0.005	0.534	1.17	94.6	3.418 ± 0.005	0.534	0.45	74.7
Rivastigmine	0.950 ± 0.020	−0.022	−0.19	39.8	1.546 ± 0.004	0.189	−0.48	25.1

**Table 10:** Biomimetic distribution-model parameters of the investigated analogues in comparison with donepezil and rivastigmine. log*k*—logarithm of the chromatographic retention factor; logK—transformed equilibrium-partition descriptor; CHI_IAM_—chromatographic hydrophobicity index based on the IAM column; logV_d_—logarithm of the model-derived volume of distribution; V_d_—model-derived volume of distribution (L/kg); logV_du_—logarithm of the model-derived unbound volume of distribution (L/kg); BTB—model-derived brain tissue binding. The V_d_, V_du_ and BTB outputs are empirical estimates and should be used primarily for comparative ranking within the applicability domain of the underlying HSA/IAM models.

Compound	log*k*_HSA_	logK_HSA_	CHI_IAM_	logK_IAM_	log*k*_IAM_	logV_d_	V_d_ (L/kg)	logV_du_	log*k*_BTB_	%BTB
(**1**)	1.549	4.71	71.9	12.773	3.729	3.96	9224.1	5.85	4.04	97.6
(**2**)	1.420	4.14	65.7	9.757	3.441	2.76	579.6	4.43	3.53	97.2
(**3**)	1.503	4.50	66.7	10.209	3.490	2.88	736.4	4.70	3.68	97.4
(**4**)	1.478	4.38	64.6	9.318	3.392	2.52	327.6	4.30	3.53	97.2
(**5**)	1.436	4.20	71.6	12.564	3.711	3.98	9619.2	5.65	3.97	97.5
(**6**)	1.425	4.16	63.0	8.701	3.317	2.29	198.8	3.98	3.38	97.1
Donepezil	1.173	3.23	45.0	4.192	2.488	0.51	3.27	1.83	2.05	95.3
Rivastigmine	−0.187	0.83	39.0	3.351	2.213	0.67	4.70	0.91	0.29	74.5

**Table 11:** First-order kinetic parameters for the plasma stability of compounds (**1**)–(**6**) in pooled human and rat plasma. The apparent first-order degradation rate constant (kobs), coefficient of determination (R^2^), regression *p* value, apparent plasma half-life (t1/2), corresponding 95% confidence interval (CI) and percentage of parent compound remaining after 24 h are reported. Linear regression was performed using the mean normalized analyte response at seven incubation time points (0, 0.5, 1, 1.5, 2, 4, and 24 h). Supplementary Information (SI) table (Tables S1–S12) refers to the corresponding table containing the underlying triplicate plasma-stability data. * For profiles in which the 95% CI of kobs included zero, the upper confidence limit of the apparent half-life could not be estimated; in these cases, only the lower 95% confidence bound is reported (≥value), and the half-life should be interpreted as an uncertain extrapolation beyond the 24 h observation period.

Compound	Matrix	SI Table	*k*_obs_ (h^−1^)	R^2^	*p*	Apparent t_1/2_ (h)	95% CI of t1/2 (h)	Remaining at 24 h (%)
(**1**)	Human	S1	0.00417	0.798	0.007	166	105–394	89.0
(**1**)	Rat	S2	0.01650	0.639	0.031	42	23–310	62.2
(**2**)	Human	S3	0.00748	0.886	0.002	93	66–158	81.4
(**2**)	Rat	S4	0.01198	0.747	0.012	58	35–175	69.3
(**3**)	Human	S5	0.00890	0.784	0.008	78	49–196	78.2
(**3**)	Rat	S6	0.00885	0.414	0.119	78	≥33 *	70.7
(**4**)	Human	S7	0.00847	0.900	0.001	82	59–133	80.8
(**4**)	Rat	S8	0.00861	0.540	0.060	81	≥39 *	75.4
(**5**)	Human	S9	0.00972	0.918	<0.001	71	53–109	77.5
(**5**)	Rat	S10	0.00566	0.534	0.062	122	≥59 *	80.5
(**6**)	Human	S11	0.00303	0.499	0.076	228	≥106 *	89.5
(**6**)	Rat	S12	0.00800	0.646	0.029	87	47–582	79.9

**Table 12:** Aqueous solubility of the investigated compounds in comparison with donepezil and rivastigmine. JP1—artificial gastric fluid (pH 1.2); JP2—artificial intestinal fluid (pH 6.8); pH 7.4—phosphate buffer representing physiological pH; *k*′—capacity (retention) factor.

Compound	JP1 [µM] ± CV [%]	JP2 [µM] ± CV [%]	7.4 [µM] ± CV [%]	*k*′± CV [%]
(**1**)	149.0 ± 4.7	128.0 ± 0.2	53.2 ± 3.0	3.50 ± 0.2
(**2**)	156.2 ± 2.6	143.4 ± 0.3	164.9 ± 4.0	3.15 ± 0.8
(**3**)	157.5 ± 3.7	142.5 ± 1.5	127.8 ± 2.7	3.36 ± 0.6
(**4**)	148.6 ± 4.7	138.5 ± 1.5	105.3 ± 2.4	3.46 ± 0.5
(**5**)	170.8 ± 1.1	169.9 ± 0.2	123.9 ± 1.4	3.20 ± 0.3
(**6**)	123.5 ± 2.1	118.8 ± 2.4	110.4 ± 0.6	3.05 ± 0.4
Donepezil	146.8 ± 1.5	145.7 ± 3.3	127.9 ± 2.7	3.00 ± 0.3
Rivastigmine	157.5 ± 1.7	165.6 ± 1.8	156.3 ± 0.2	2.64 ± 0.2

## Data Availability

The original contributions presented in this study are included in the article/Supplementary Materials.

## Supplementary Materials

The following supporting information can be downloaded at https://www.mdpi.com/article/10.3390/membranes16100315/s1, Table S1: Results of the incubation of compound (**1**) in human plasma over 24 h; Table S2: Results of the incubation of compound (**1**) in rat plasma over 24 h; Table S3: Results of the incubation of compound (**2**) in human plasma over 24 h; Table S4: Results of the incubation of compound (**2**) in rat plasma over 24 h; Table S5: Results of the incubation of compound (**3**) in human plasma over 24 h; Table S6: Results of the incubation of compound (**3**) in rat plasma over 24 h; Table S7: Results of the incubation of compound (**4**) in human plasma over 24 h; Table S8: Results of the incubation of compound (**4**) in rat plasma over 24 h; Table S9: Results of the incubation of compound (**5**) in human plasma over 24 h; Table S10: Results of the incubation of compound (**5**) in rat plasma over 24 h; Table S11: Results of the incubation of compound (**6**) in human plasma over 24 h; Table S12: Results of the incubation of compound (**6**) in rat plasma over 24 h.
