# Supplementary material for: Biomimetic ADME Profiling of Multifunctional Biphenylalkoxyamine-Related Histamine H3 Receptor Ligands with Anti-Alzheimer Potential
Source: Membranes (Basel). 2026 Sep 25;16(10):315. doi: 10.3390/membranes16100315 (PMC13706960; doi:10.3390/membranes16100315)
Supplement: Supplementary file 1 [file membranes-16-00315-s001.zip › membranes-4534193-supplementary.pdf]

Supporting Information

Biomimetic ADME Profiling of Multifunctional Biphenylalkoxyamine Histamine H_3 Receptor Ligands with Anti-Alzheimer Potential

Stefan Simić [1,2,*], Predrag Kalaba [1], Judith Wackerlig [1], Dorota Łażewska [3], Thierry Langer [1,*]

[1]Department of Pharmaceutical Sciences, University of Vienna, Josef-Holaubek-Platz 2, 1090 Vienna, Austria

[2]Vienna Doctoral School of Pharmaceutical, Nutritional and Sport Sciences, University of Vienna, Josef-Holaubek-Platz 2, 1090 Vienna, Austria

[3]Department of Technology and Biotechnology of Drugs, Jagiellonian University Medical College, Medyczna Str. 9, 30-688 Kraków, Poland

* Correspondance to:

Thierry Langer

Department of Pharmaceutical Sciences, Faculty of Life Sciences, University of Vienna, Josef-Holaubek-Platz 2, Vienna, Austria

E-Mail: thierry.langer@univie.ac.at

and

Stefan Simić

Department of Pharmaceutical Sciences, Faculty of Life Sciences, University of Vienna, Josef-Holaubek-Platz 2, Vienna, Austria

E-Mail: stefan.simic@univie.ac.at

Table S1. Results of the incubation of (**1**) in human plasma over 24.00 h. Three samples were incubated over 24.00 h and measured with MS. The detected areas of (**1**) peaks were divided by the detected area of IS peaks (losartan). The average of the same time was converted to A(Compound)/A(Standard). The calculated values were referred to the recovered peak area at t = 0.00 h to receive the percentage of the analyte (n = 3).

	Peak area						
**Time [h]**	**(1)**	**Donepezil**	**A(Compound)/A(Standard)**	**A/A mean**	**Standard dev.**	**Var. [%]**	**Unmodified [%]**
0	93037312	134691152	0.691				
0	93633016	139302304	0.672	0.673	0.017	2.5	100.0
0	81242320	123755520	0.656				
0.5	92732880	134352944	0.690				
0.5	77205784	122250784	0.632	0.666	0.031	4.6	99.0
0.5	79360832	117169768	0.677				
1	82204064	118105040	0.696				
1	76361792	121140624	0.630	0.663	0.033	5.0	98.4
1	80948264	122354360	0.662				
1.5	84121720	122993408	0.684				
1.5	90830392	141507040	0.642	0.651	0.030	4.6	96.6
1.5	90395216	144446176	0.626				
2	77668512	120859816	0.643				
2	76915672	123414088	0.623	0.642	0.019	3.0	95.4
2	80948264	122354360	0.662				
4	74617600	118132032	0.632				
4	80885296	125324064	0.645	0.629	0.018	2.8	93.5
4	76361792	125072344	0.611				
24	68088072	121580496	0.560				
24	72992664	114789000	0.636	0.599	0.038	6.3	89.0
24	73186648	121512112	0.602				

Table S2. Results of the incubation of (**1**) in rat plasma over 24.00 h. Three samples were incubated over 24.00 h and measured with MS. The detected areas of (**1**) peaks were divided by the detected area of IS peaks (losartan). The average of the same time was converted to A(Compound)/A(Standard). The calculated values were referred to the recovered peak area at t = 0.00 h to receive the percentage of the analyte (n = 3).

	**Peak area**						
**Time [h]**	**(1)**	**Donepezil**	**A(Compound)/A(Standard)**	**A/A mean**	**Standard dev.**	**Var. [%]**	**Unmodified [%]**
0	91210352	105160824	0.867				
0	102981752	112352680	0.917	0.885	0.027	3.1	100.0
0	99367688	113974136	0.872				
0.5	96490152	119168880	0.810				
0.5	91633504	113001432	0.811	0.832	0.037	4.5	94.0
0.5	97567200	111560376	0.875				
1	94409544	112697392	0.838				
1	86441048	106002168	0.815	0.808	0.035	4.3	91.2
1	91020752	118281128	0.770				
1.5	81433912	109400688	0.744				
1.5	90418592	111862480	0.808	0.792	0.042	5.4	89.5
1.5	91805904	111322384	0.825				
2	77919248	110050744	0.708				
2	82280840	119327032	0.690	0.714	0.028	3.9	80.7
2	85071536	114254160	0.745				
4	70334296	123274280	0.571				
4	73470120	119805752	0.613	0.600	0.026	4.3	67.8
4	76049536	123307968	0.617				
24	68669392	124919192	0.550				
24	67272016	122938456	0.547	0.551	0.005	0.8	62.2
24	68876856	123849168	0.556				

Table S3. Results of the incubation of (**2**) in human plasma over 24.00 h. Three samples were incubated over 24.00 h and measured with MS. The detected areas of (**2**) peaks were divided by the detected area of IS peaks (losartan). The average of the same time was converted to A(Compound)/A(Standard). The calculated values were referred to the recovered peak area at t = 0.00 h to receive the percentage of the analyte (n = 3).

	Peak area						
**Time [h]**	**(2)**	**Donepezil**	**A(Compound)/A(Standard)**	**A/A mean**	**Standard dev.**	**Var. [%]**	**Unmodified [%]**
0	78092816	139935872	0.558				
0	66765224	119449448	0.559	0.568	0.016	2.8	100.0
0	83468576	142325920	0.586				
0.5	76099728	130469920	0.583				
0.5	64105980	117619624	0.545	0.561	0.020	3.5	98.8
0.5	74189016	133767248	0.555				
1	70070000	125258864	0.559				
1	63843540	120273056	0.531	0.546	0.014	2.6	96.2
1	67338040	122657152	0.549				
1.5	69521800	127187024	0.547				
1.5	63147208	118810928	0.531	0.536	0.009	1.8	94.4
1.5	67449968	127456224	0.529				
2	67383160	125923352	0.535				
2	60812912	114043776	0.533	0.525	0.016	3.0	92.5
2	59642628	117713056	0.507				
4	62051948	116344104	0.533				
4	58616484	115813608	0.506	0.523	0.015	2.9	92.2
4	64758372	122037656	0.531				
24	64511420	139553200	0.462				
24	60924824	131598504	0.463	0.462	0.001	0.2	81.4
24	57733144	125162472	0.461				

Table S4. Results of the incubation of (**2**) in rat plasma over 24.00 h. Three samples were incubated over 24.00 h and measured with MS. The detected areas of (**2**) peaks were divided by the detected area of IS peaks (losartan). The average of the same time was converted to A(Compound)/A(Standard). The calculated values were referred to the recovered peak area at t = 0.00 h to receive the percentage of the analyte (n = 3).

	**Peak area**						
**Time [h]**	**(2)**	**Donepezil**	**A(Compound)/A(Standard)**	**A/A mean**	**Standard dev.**	**Var. [%]**	**Unmodified [%]**
0	72008288	114355536	0.630				
0	73975920	117893304	0.627	0.625	0.006	1.0	100.0
0	68380296	110652984	0.618				
0.5	67669992	114101816	0.593				
0.5	71108048	121310224	0.586	0.597	0.013	2.1	95.4
0.5	73178032	119868112	0.610				
1	62124244	108290320	0.574				
1	65191700	116794328	0.558	0.552	0.025	4.5	88.4
1	59541496	113424048	0.525				
1.5	64627096	119895144	0.539				
1.5	61994728	115658024	0.536	0.534	0.006	1.1	85.4
1.5	61515744	116673416	0.527				
2	65383960	123575032	0.529				
2	62995384	119670368	0.526	0.529	0.002	0.5	84.6
2	63579984	119656888	0.531				
4	60622808	120264296	0.504				
4	55504364	114215992	0.486	0.505	0.020	4.0	80.8
4	62012080	117891304	0.526				
24	53615656	125775152	0.426				
24	57940036	133942968	0.433	0.433	0.007	1.6	69.3
24	55519956	126251368	0.440				

Table S5. Results of the incubation of (**3**) in human plasma over 24.00 h. Three samples were incubated over 24.00 h and measured with MS. The detected areas of (**3**) peaks were divided by the detected area of IS peaks (losartan). The average of the same time was converted to A(Compound)/A(Standard). The calculated values were referred to the recovered peak area at t = 0.00 h to receive the percentage of the analyte (n = 3).

	Peak area						
**Time [h]**	**(3)**	**Donepezil**	**A(Compound)/A(Standard)**	**A/A mean**	**Standard dev.**	**Var. [%]**	**Unmodified [%]**
0	81378696	115671672	0.704				
0	80434584	112020224	0.718	0.701	0.019	2.7	100.0
0	75256120	110556136	0.681				
0.5	83665768	116422096	0.719				
0.5	81881272	119551704	0.685	0.695	0.021	3.0	99.1
0.5	80225416	117853304	0.681				
1	79682144	117579808	0.678				
1	82817688	114785488	0.721	0.686	0.032	4.6	97.9
1	76342728	115677560	0.660				
1.5	66657384	109364184	0.609				
1.5	83416176	124586072	0.670	0.636	0.031	4.8	90.7
1.5	69365640	110407968	0.628				
2	61219944	99569872	0.615				
2	73334992	112260352	0.653	0.634	0.019	3.0	90.5
2	71484304	112813032	0.634				
4	69716048	112498216	0.620				
4	67281080	111907528	0.601	0.620	0.018	2.9	88.4
4	74267544	116469872	0.638				
24	60258392	113735528	0.530				
24	62164200	111935760	0.555	0.548	0.016	2.9	78.2
24	59387752	106219624	0.559				

Table S6. Results of the incubation of (**3**) in rat plasma over 24.00 h. Three samples were incubated over 24.00 h and measured with MS. The detected areas of (**3**) peaks were divided by the detected area of IS peaks (losartan). The average of the same time was converted to A(Compound)/A(Standard). The calculated values were referred to the recovered peak area at t = 0.00 h to receive the percentage of the analyte (n = 3).

	Peak area						
**Time [h]**	**(3)**	**Donepezil**	**A(Compound)/A(Standard)**	**A/A mean**	**Standard dev.**	**Var. [%]**	**Unmodified [%]**
0	66272376	109976928	0.603				
0	72692456	118167008	0.615	0.626	0.030	4.7	100.0
0	73578352	111672192	0.659				
0.5	66057396	121275112	0.545				
0.5	64906632	119589456	0.543	0.553	0.016	2.9	88.4
0.5	69141904	121054968	0.571				
1	60917740	114371144	0.533				
1	59252336	113065256	0.524	0.529	0.004	0.8	84.5
1	62595200	118272528	0.529				
1.5	61886736	118587632	0.522				
1.5	58054412	118076336	0.492	0.496	0.024	4.8	79.3
1.5	53244100	112166392	0.475				
2	59083360	116408000	0.508				
2	59244124	120171272	0.493	0.492	0.017	3.4	78.6
2	56809640	119779064	0.474				
4	56106484	117252424	0.479				
4	53269516	120928120	0.441	0.457	0.019	4.2	73.1
4	52450204	115874880	0.453				
24	29853746	67695456	0.441				
24	29738580	67699456	0.439	0.442	0.003	0.8	70.7
24	29951662	67182840	0.446				

Table S7. Results of the incubation of (**4**) in human plasma over 24.00 h. Three samples were incubated over 24.00 h and measured with MS. The detected areas of (**4**) peaks were divided by the detected area of IS peaks (losartan). The average of the same time was converted to A(Compound)/A(Standard). The calculated values were referred to the recovered peak area at t = 0.00 h to receive the percentage of the analyte (n = 3).

	Peak area						
**Time [h]**	**(4)**	**Donepezil**	**A(Compound)/A(Standard)**	**A/A mean**	**Standard dev.**	**Var. [%]**	**Unmodified [%]**
0	86244344	113818848	0.758				
0	80565184	110444472	0.729	0.738	0.017	2.3	100.0
0	79944256	109780024	0.728				
0.5	82907728	115422544	0.718				
0.5	88124896	118513592	0.744	0.734	0.014	1.8	99.4
0.5	86989392	117683280	0.739				
1	82199976	114040016	0.721				
1	83904048	115394744	0.727	0.729	0.009	1.2	98.7
1	90415080	122550064	0.738				
1.5	86765176	117749560	0.737				
1.5	81488336	115441376	0.706	0.714	0.020	2.7	96.7
1.5	83725200	119506320	0.701				
2	81724240	114395688	0.714				
2	80028608	112767912	0.710	0.711	0.003	0.4	96.3
2	81319288	114571680	0.710				
4	74572576	113286280	0.658				
4	80650992	115687568	0.697	0.666	0.029	4.3	90.1
4	72547912	113159808	0.641				
24	61912536	108641880	0.570				
24	70203616	114212808	0.615	0.596	0.024	3.9	80.8
24	68962784	114023616	0.605				

Table S8. Results of the incubation of (**4**) in rat plasma over 24.00 h. Three samples were incubated over 24.00 h and measured with MS. The detected areas of (**4**) peaks were divided by the detected area of IS peaks (losartan). The average of the same time was converted to A(Compound)/A(Standard). The calculated values were referred to the recovered peak area at t = 0.00 h to receive the percentage of the analyte (n = 3).

	Peak area						
**Time [h]**	**(4)**	**Donepezil**	**A(Compound)/A(Standard)**	**A/A mean**	**Standard dev.**	**Var. [%]**	**Unmodified [%]**
0	80928904	117689728	0.688				
0	76086216	116683096	0.652	0.659	0.026	3.9	100.0
0	75461592	118298912	0.638				
0.5	71070832	112732504	0.630				
0.5	70557480	116396752	0.606	0.621	0.013	2.1	94.2
0.5	73177080	116699360	0.627				
1	75875616	116897248	0.649				
1	69882536	116069760	0.602	0.618	0.027	4.4	93.7
1	70744776	117495640	0.602				
1.5	66928180	120612072	0.555				
1.5	69308144	122594056	0.565	0.566	0.012	2.1	85.9
1.5	72477880	125269296	0.579				
2	66044808	125331024	0.527				
2	66450344	118618024	0.560	0.550	0.020	3.6	83.4
2	69116888	122921960	0.562				
4	66337008	124554656	0.533				
4	60197912	117051720	0.514	0.527	0.011	2.1	80.0
4	64942284	121500328	0.535				
24	60624424	122532432	0.495				
24	59114688	118423592	0.499	0.497	0.002	0.4	75.4
24	55879776	112540104	0.497				

Table S9. Results of the incubation of (**5**) in human plasma over 24.00 h. Three samples were incubated over 24.00 h and measured with MS. The detected areas of (**5**) peaks were divided by the detected area of IS peaks (losartan). The average of the same time was converted to A(Compound)/A(Standard). The calculated values were referred to the recovered peak area at t = 0.00 h to receive the percentage of the analyte (n = 3).

	**Peak area**						
**Time [h]**	**(5)**	**Donepezil**	**A(Compound)/A(Standard)**	**A/A mean**	**Standard dev.**	**Var. [%]**	**Unmodified [%]**
0	76833368	126719472	0.606				
0	70529136	112574288	0.627	0.609	0.017	2.8	100.0
0	69591064	117406072	0.593				
0.5	66755128	114231080	0.584				
0.5	69095176	116216272	0.595	0.597	0.013	2.2	98.0
0.5	70986112	116216272	0.611				
1	67255360	111228824	0.605				
1	65431249	110855600	0.590	0.595	0.009	1.4	97.7
1	68941144	116986328	0.589				
1.5	63149404	111932376	0.564				
1.5	72842632	123978864	0.588	0.582	0.015	2.7	95.6
1.5	73288192	123509080	0.593				
2	67997160	119677872	0.568				
2	61707516	112394440	0.549	0.564	0.013	2.3	92.6
2	65432168	114021120	0.574				
4	63945920	120862288	0.529				
4	63459412	110893008	0.572	0.546	0.023	4.2	89.8
4	60319364	112292656	0.537				
24	59450352	119194192	0.499				
24	53280564	116399304	0.458	0.472	0.023	5.0	77.5
24	52325692	114101504	0.459				

Table S10. Results of the incubation of (**5**) in rat plasma over 24.00 h. Three samples were incubated over 24.00 h and measured with MS. The detected areas of (**5**) peaks were divided by the detected area of IS peaks (losartan). The average of the same time was converted to A(Compound)/A(Standard). The calculated values were referred to the recovered peak area at t = 0.00 h to receive the percentage of the analyte (n = 3).

	Peak area						
**Time [h]**	**(5)**	**Donepezil**	**A(Compound)/A(Standard)**	**A/A mean**	**Standard dev.**	**Var. [%]**	**Unmodified [%]**
0	56218268	111683400	0.503				
0	58423264	115992776	0.504	0.509	0.009	1.8	100.0
0	64767348	124716800	0.519				
0.5	54320884	118735864	0.457				
0.5	63001396	137180528	0.459	0.465	0.011	2.3	91.3
0.5	51434212	107821656	0.477				
1	50795392	110568288	0.459				
1	53301768	115585296	0.461	0.456	0.008	1.8	89.6
1	50415872	112921464	0.446				
1.5	50778068	117841096	0.431				
1.5	51309672	114984008	0.446	0.448	0.018	4.1	88.1
1.5	52102116	111440896	0.468				
2	51095532	113938960	0.448				
2	50556704	115634592	0.437	0.445	0.007	1.5	87.4
2	53295468	118731672	0.449				
4	51089632	121243840	0.421				
4	51848044	118963512	0.436	0.434	0.011	2.6	85.2
4	53051948	119565592	0.444				
24	51765408	123361416	0.420				
24	45857096	111152632	0.413	0.409	0.012	3.0	80.5
24	46220564	116726080	0.396				

Table S11. Results of the incubation of (**6**) in human plasma over 24.00 h. Three samples were incubated over 24.00 h and measured with MS. The detected areas of (**6**) peaks were divided by the detected area of IS peaks (losartan). The average of the same time was converted to A(Compound)/A(Standard). The calculated values were referred to the recovered peak area at t = 0.00 h to receive the percentage of the analyte (n = 3).

	Peak area						
**Time [h]**	**(6)**	**Donepezil**	**A(Compound)/A(Standard)**	**A/A mean**	**Standard dev.**	**Var. [%]**	**Unmodified [%]**
0	62025184	117476392	0.528				
0	72554104	128961936	0.563	0.548	0.018	3.3	100.0
0	69351976	125322736	0.553				
0.5	68070464	127688792	0.533				
0.5	75965064	137725392	0.552	0.527	0.028	5.4	96.1
0.5	61240028	123537400	0.496				
1	63866440	121643008	0.525				
1	67781040	131819528	0.514	0.523	0.008	1.6	95.5
1	64293052	121174008	0.531				
1.5	62858792	124554896	0.505				
1.5	71016904	138145248	0.514	0.520	0.018	3.5	94.8
1.5	70842600	131180232	0.540				
2	72537184	147756368	0.491				
2	76372656	145821968	0.524	0.508	0.016	3.2	92.6
2	72233256	142225872	0.508				
4	64108784	133245656	0.481				
4	68112904	136600288	0.499	0.499	0.018	3.7	91.1
4	70559088	136248624	0.518				
24	71856544	138116288	0.520				
24	61353728	129590096	0.473	0.491	0.026	5.2	89.5
24	63096660	131908752	0.478				

Table S12. Results of the incubation of (**6**) in rat plasma over 24.00 h. Three samples were incubated over 24.00 h and measured with MS. The detected areas of (**6**) peaks were divided by the detected area of IS peaks (losartan). The average of the same time was converted to A(Compound)/A(Standard). The calculated values were referred to the recovered peak area at t = 0.00 h to receive the percentage of the analyte (n = 3).

	**Peak area**						
**Time [h]**	**(6)**	**Donepezil**	**A(Compound)/A(Standard)**	**A/A mean**	**Standard dev.**	**Var. [%]**	**Unmodified [%]**
0	65114740	120338736	0.541				
0	63727604	122166104	0.522	0.537	0.014	2.6	100.0
0	68407136	124551952	0.549				
0.5	71163648	128179952	0.555				
0.5	69393360	131242088	0.529	0.535	0.018	3.4	99.5
0.5	64961856	124746824	0.521				
1	62056208	122970408	0.505				
1	66537884	126563008	0.526	0.516	0.011	2.1	96.1
1	66477472	128352080	0.518				
1.5	60493112	126512928	0.478				
1.5	62826004	124615744	0.504	0.497	0.016	3.2	92.4
1.5	63475456	125060600	0.508				
2	62835664	126678184	0.496				
2	63891016	126963840	0.503	0.492	0.013	2.6	91.7
2	57872580	121012912	0.478				
4	57584092	126894264	0.454				
4	65746736	138964032	0.473	0.450	0.026	5.8	83.7
4	53624320	127120168	0.422				
24	54257436	124901896	0.434				
24	54315900	123651064	0.439	0.429	0.014	3.2	79.9
24	51878352	125374584	0.414				
